# Combined Effects of Biochar and Wood Distillate on Growth, Yield, and Fruit Quality of Soilless-Grown Highbush Blueberry Plants (*Vaccinium corymbosum* L.)

**DOI:** 10.3390/plants14243773

**Published:** 2025-12-11

**Authors:** Anna Agosti, Samreen Nazeer, Leandra Leto, Jasmine Hadj Saadoun, Alessia Levante, Elena Maestri, Martina Cirlini, Benedetta Chiancone

**Affiliations:** 1 Department of Food and Drug, University of Parma, Viale Parco Area delle Scienze 27/A, 43124 Parma, Italy; anna.agosti@unipr.it (A.A.); samreen.nazeer@unipr.it (S.N.); leandra.leto@unipr.it (L.L.); jasmine.hadjsaadoun@unipr.it (J.H.S.); alessia.levante@unipr.it (A.L.); martina.cirlini@unipr.it (M.C.); 2 Department of Chemistry, Life Sciences and Environmental Sustainability, University of Parma, Parco Area delle Scienze 11/A, 43124 Parma, Italy; elena.maestri@unipr.it; 3 Institute of Biophysics, National Research Council (CNR), Via Ugo La Malfa 153, 90146 Palermo, Italy

**Keywords:** biochar, blueberry, soilless, substrate, wood distillate

## Abstract

The global production of blueberries (*Vaccinium corymbosum* L.) has increased rapidly due to rising demand for antioxidant-rich fruits, making this crop increasingly important worldwide. Because blueberries require acidic soils, soilless systems offer a promising alternative by optimizing nutrient availability and reducing soil-related limitations. Among sustainable amendments, biochar (BC) improves water retention, porosity, and microbial activity, while wood distillate (WD), rich in bioactive compounds, can enhance plant resilience and growth. Although often used separately, their combined application may exert synergistic effects on substrate fertility and plant performance. This study investigated the effects of BC and WD, alone and in combination, on the growth, yield, and fruit quality of the ‘Cargo’ blueberry cultivar grown in a soilless system. Two distinct harvests were conducted during the growing season, and statistical analyses were performed independently for each, assessing treatment effects in relation to harvest timing. Moreover, the metabolic activity of the substrate’s microbial community was evaluated to assess the impact of the treatments. Results showed that BC application, particularly at 10%, significantly enhanced plant yield and fruit quality, increasing total phenolic content and antioxidant activity, while WD exhibited variable, dose-dependent effects on growth and biochemical traits, highlighting species-specific responses in soilless blueberry cultivation.

## 1. Introduction

Global blueberry plant production (*Vaccinium corymbosum* L.) has more than doubled over the past decade, exceeding 1 million tonnes in 2023 driven by the rising consumer demand for antioxidant-rich fruits. Blueberry cultivation has also expanded significantly in Italy, where production has reached 9000 tonnes annually [1,2]. Despite this growth, the species’ strict requirement for acidic soils (pH 4.0–5.5) represents a major limitation for conventional field production [1,3,4,5]. Farmers often rely on costly soil-acidification practices such as sulfur application to achieve suitable pH levels [6,7]. These constraints have stimulated interest in alternative production systems, such as container or soilless cultivation, which offer more precise control of substrate properties and the possibility to mitigate soil-related limitations [8,9,10]. Soilless systems provide several advantages, including improved rhizosphere conditions, optimized nutrient availability, better water regulation, and reduced exposure to abiotic stresses [11]. These factors support vigorous root development and can enhance early productivity [12]. Coconut fiber is one of the most widely used soilless substrates, particularly in Europe and in the Western United States [13]. Coconut fiber offers multiple benefits for blueberry cultivation, but it also presents some challenges; for instance, its high water-holding capacity can negatively affect the air-water balance, reducing oxygen diffusion to the roots [14]. Additionally, concerns have been raised about its environmental impact due to land use during coconut harvesting [15].

To address these limitations, the incorporation of coarser materials into coconut fiber has been proposed to improve substrate aeration [14]. One promising material that could be used in combination with coconut fiber is biochar (BC), a sustainable amendment that has attracted significant attention in recent years [16]. BC is a by-product of pyrolysis, a thermochemical process conducted in the absence of oxygen from plant materials such as pruning residues and wood waste [17,18]. BC offers several advantages for plant cultivation, including improving soil water and nutrient retention, enhancing substrate porosity and oxygen permeability, and promoting the growth of beneficial microorganisms and mycorrhizal fungi due to its large surface area [19,20]. Although biochar has been studied extensively in many crops, its application in small fruit species remains limited [21,22]. Nonetheless, interest among growers is increasing, as BC may improve yield and profitability in high-value small fruit production [23]. Several studies have already demonstrated the potential of BC in blueberry cultivation, both in open-field and soilless systems, highlighting its effectiveness as an amendment, whether used alone or in combination with biostimulants [20,24,25]. Wood distillate (WD), another pyrolysis-derived product, contains numerous bioactive compounds, including esters, alcohols, sugars, acids, and phenols [26]. WD has been shown to enhance plant tolerance to biotic and abiotic stresses, stimulate growth, improve soil conditions in acidic substrates, increase nutrient availability, reduce nitrogen loss, and positively modulate soil microflora [25,27]. In particular, WD can increase sugar accumulation by improving soil nutritional quality and improving nutrient uptake, especially NH_4_^+^, Mg^2+^, K^+^, and Fe^2+^, with magnesium playing a crucial role in carbohydrate metabolism. Evidence also suggests that combined application of BC and WD may exert synergistic effects on soil fertility and plant performance [25,28].

This study aimed to evaluate the individual and combined effects of BC and WD on the growth, total yield, and fruit quality of the ‘Cargo’ highbush blueberry cultivar grown in a soilless system. Furthermore, the study investigated how these products, applied alone or together, influence the functional activity of the substrate’s microbial community. Because fruit quality traits can vary markedly across the harvesting season [29], the two harvest dates were analyzed separately to capture temporal dynamics in treatment responses.

## 2. Results and Discussion

To the best of authors’ knowledge, the use of biochar as partial substitute for coconut fiber in a blueberry soilless cultivation system, combined with the application of wood distillate, has not yet been investigated. Although the long-term positive effects of biochar on plant performance are well documented, the novelty of this investigation justifies the presentation of first year results. Moreover, to better isolate the effect of biochar, as a sustainable substrate component, wood distillate was applied at a single concentration, consistent with the only previously published study evaluating their combined use on blueberry plants, in an open field trial [25]. Because blueberry harvest typically extends over 5–7 weeks, during which fruit attributes can change substantially [30], this study assessed fruit quality at multiple time points. This approach allowed us to capture the dynamic nature of fruit maturation and to define best practices to minimize postharvest losses and to preserve quality through frequent harvests [31].

### 2.1. Plant Growth and Physiological Status

Before discussing the results related to fruit quality parameters, it is important to present data on plant status following biochar application.

Between the vegetative and the flowering stages, BC did not statistically affect the plant growth, as branch height remained comparable across treatments (4 ± 1 cm for BC0 vs. 8 ± 2 cm for BC-treated plants, on average; *p* = 0.328). From flowering to harvest, however, a slight but significant effect of the “WD Treatment” was observed, regardless of biochar presence (*p* = 0.046), with WD-treated plants developing shorter branches (7 ± 2 cm for WD vs. 15 ± 3 cm for 0WD). These results confirm, on one hand, that biochar can be used as a partial substrate replacement without negatively affecting plant growth [32], and on the other, that the plant response to WD is highly genotype- and dose-dependent [33].

Non-destructive measurements performed before harvest provided additional insights into the physiological condition of the plants under each treatment (Table S1). Flavonoids (Flv), which accumulate in leaves and play a key role in plant defense against environmental and biotic stresses [34], did not vary significantly among treatments, suggesting that plant health remained stable under all examined conditions (Table S1).

SPAD value, widely used as a valuable parameter for the rapid assessment of chlorophyll content and nitrogen status in crops [35,36], also showed no significant treatment effects. The literature reports conflicting results regarding the effect of biochar on SPAD. For example, Chrysargyris et al. [37] reported a decrease in SPAD values as the biochar concentration in the substrate increased for *Antirrhinum majus*, instead Ren et al. [38] demonstrated that, in tobacco, increasing biochar concentration in the substrate led to higher SPAD values in the leaves. Moreover, Hafez et al. [39] reported an increase in SPAD values when WD treatment was performed in canola. In the present study, the statistical analysis revealed no statistically significant differences in SPAD values of blueberry leaves prior to harvest. Neither the “BC Treatment” nor the “WD Treatment” factors had any effect on SPAD values (Table S1).

Chlorophyll fluorescence is a key photosynthetic parameter that reflects light absorption and utilization in PSII, with Fv/Fm indicating the efficiency of primary light energy conversion in its reaction center [40]. In this study, the two-way ANOVA performed considering the factors “BC Treatment” and “WD Treatment” before harvest, showed that the maximum quantum efficiency of PSII photochemistry (Fv/Fm) was not significantly affected by both BC and WD treatments (Table S1). This is consistent with Li et al. [41] who reported no effect of 0.5–2% biochar on Fv/Fm in cucumber plants, although it contrasts with findings by Liu et al. [42], who observed increased Fv/Fm tomato following biochar amendment.

**Figure 1:**
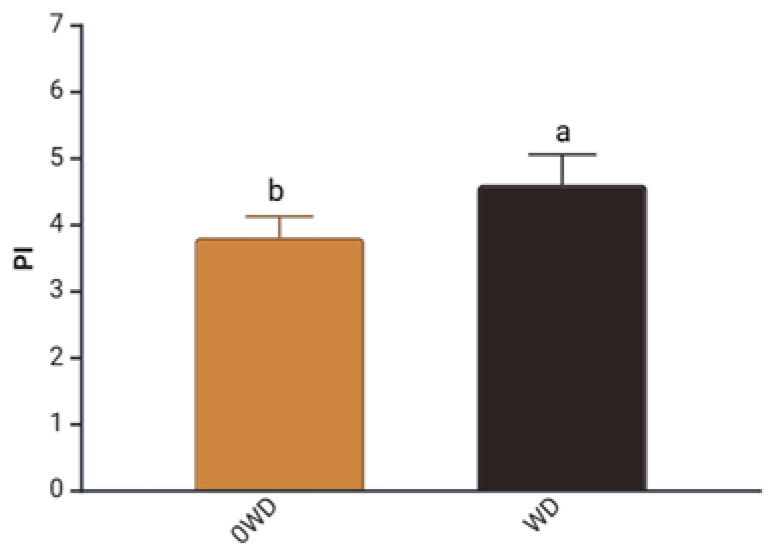
Effect of “WD Treatment” on Performance Index (PI) at harvest time. Two-way ANOVA, different letters indicate sig-nificantly different values at *p* ≤ 0.05 according to Tukey’s test. 0WD: without wood distillate, WD: treatment with wood distillate.

A significant effect of “WD Treatment” was, however, detected for the Performance Index (PI) measured before fruit harvest to assess their physiological status (Table S1). PI integrates key components such as the density of active reaction centers (RCs), the efficiency of electron transport, and the probability of photon capture by RCs [43]. WD-treated plants showed, on average, a 32% increase in PI prior to harvest (Figure 1), consistent with Fedeli et al. [44], who reported that *Lactuca sativa* (L.) grown under hydroponic conditions exhibited an increase in PI, following WD application.

### 2.2. Substrate Chemical Properties

It is noteworthy to consider the effect of biochar and wood distillate treatments on substrate pH and electrical conductivity. Blueberry is highly sensitive to soil pH, with optimal growth occuring under acidic conditions (pH 4.5–5.5) [45]. Although pH increased following the treatments, particularly in plants with 10% biochar (Table 1), the vegetative performance and subsequent yield were not negatively affected. This suggests that the treatments did not compromise plant development, even under slightly less favorable pH conditions.

### 2.3. Yield Response to Biochar and Wood Distillate

Statistical analysis of the total plant yield, evaluated at the end of both harvest dates, revealed a significant interaction between the two considered factors “BC Treatment” and “WD Treatment” (Table 2). On average, when the plants were not treated with WD, increasing concentrations of biochar was associated with higher production, respectively +26% with BC5 and +45% with BC10. The increased production observed with the BC10 treatment was also maintained in WD-treated plants, which still showed an average yield increase of 18% relative to untreated ones. The yield enhancement observed in biochar-treated plants, as reported by Li et al. [46], may be attributed to improved nutrient availability, enhanced root development, and more efficient nutrient uptake, ultimately leading to higher yields. However, the literature provides mixed evidence regarding the impact of biochar on yield. For example, Vaccari et al. [47] reported that the addition of BC has a positive effect on growth, but not on yield; Zhang et al. [25] reported that BC, produced through slow pyrolysis using wood waste, does not affect either the growth or yield of blueberry plants, while, Sales et al. [20] stated that biochar improved yield only starting from the second year after its application. The present study revealed that biochar application, particularly at 10%, had a positive effect on total production even during the first harvest season. Plants treated with BC10 recorded the highest yields, both in the absence (1350 g) and presence of WD (1094 g), significantly outperforming the BC0-0WD control (Table 2). In contrast, yield in BC5-treated plants decreased when WD was applied, with a 29% reduction relative to BC5 without WD. A similar negative effect was observed for BC10 plants, where WD application reduced yield by 19% (Table 2). In fact, WD application reduced yield particularly in BC-treated plants, with decreases of 29% in BC5 and 19% in BC10 compared with their respective untreated controls. The reduction in yield associated with WD treatment may be related to the dose-dependent effects. Zulkarami et al. [48] reported that a 30% WD solution caused complete plant mortality in rockmelon, while a 10% concentration improved both plant growth and yield, highlighting a narrow threshold between beneficial and inhibitory doses.

### 2.4. Fruit Physical Characteristics

Biometric parameters, such as fruit weight and size, are key physical indicators that influence overall fruit quality [29].

#### 2.4.1. Fruit Weight

The analysis of fruit weight revealed a significant effect of the “Harvest date”, other than “BC Treatment” and “WD Treatment” with a notable decrease, approximately 27%, observed between the 1st and 2nd harvest, regardless of treatment or WD application (Figure S1). A similar declining trend in fruit weight over the harvest season was also reported by Castrejon et al. [49].

To explore the specific effects of “BC Treatment” and “WD Treatment” across different harvests, each fruit quality parameter was analyzed separately using a two-way ANOVA for each harvest date. During the 1st harvest, no significant effects of either factor, nor their interaction, were observed on fruit weight. During the 2nd harvest, the two-way ANOVA revealed a statistically significant interaction between “BC Treatment” and “WD Treatment”. In particular, fruit weight was significantly higher in BC5 plants in the absence of WD compared to BC10 under the same conditions. Additionally, in WD-treated plants, BC5 showed a significant increase in fruit weight compared to BC0 (Table 3). Although not statistically significant, fruits harvested from BC10-treated plants in the absence of WD tended to have higher weights than those from other treatments. These findings contrast with those of Iqbal et al. [50], who reported a significant increase in mango fruit weight, emphasizing the beneficial role of biochar compared to untreated plants. Furthermore, this result diverges from previous studies suggesting that WD can enhance the weight of edible plant parts [48,51].

Moreover, the lack of significant differences in fruit weight among the untreated groups suggests that the increased yield observed in BC10-treated plants may be attributed to a higher number of harvested fruits.

#### 2.4.2. Fruit Size

Fruit size is economically important, as larger fruits are easier to sell, more appealing to consumers, and simpler to harvest; therefore, producing larger blueberries represents a key market objective [29,30].

Statistical analysis of fruit caliper revealed an interaction among all the examined factors (“BC Treatment”, “WD Treatment” and “Harvest date”), with an average reduction of approximately 10% in fruit size from the first to the second harvest (Figure S2). A decline in fruit size as the harvest period progresses has also been reported in several previous studies [52,53,54].

During the first harvest, the two-way ANOVA did not reveal any significant effects of either “BC Treatment” or “WD Treatment” on fruit caliper (Table 3). In contrast, in the second harvest date, an interaction between the two factors emerged (Table 3). Among all treatments, the largest fruits were obtained from BC10-treated plants. A significant increase in fruit size was observed when comparing BC5 and BC10 treated plants, in the absence of WD. Additionally, fruit weight increased in BC5-treated plants, regardless of WD application. These findings differ from those of Agosti et al. [32], who reported no changes in fruit size when tomato plants were treated with the same products, suggesting that the response of fruit size to BC and WD may be species-specific.

### 2.5. Chemical and Sensory Quality Attributes

#### 2.5.1. Total Soluble Solids (TSS)

**Figure 2:**
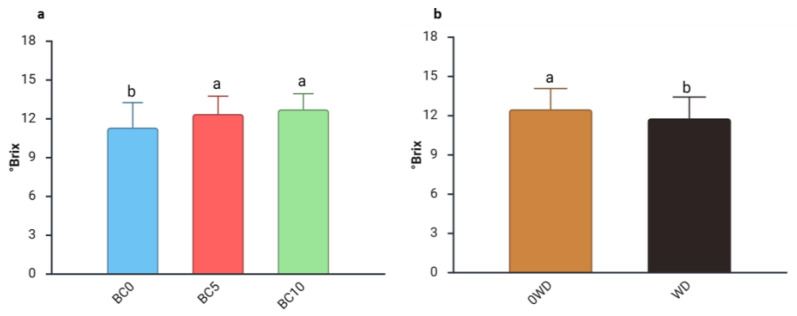
Effect of (**a**) Harvest date and (**b**) WD Treatment on blueberry fruit Total Soluble Solids (°Brix) in the 2nd harvest. Two-way ANOVA, different letters indicate significantly different values at *p* ≤ 0.05 according to Tukey’s test. BC0: 100% Coconut Fiber-CF; BC5: 5% of biochar and 95% of CF (*v*/*v*); BC10: 10% of biochar and 90% of CF (*v*/*v*); 0WD: without wood distillate, WD: treatment with wood distillate.

Fruit selection is primarily driven by appearance, followed by other sensory qualities like sugar content [55]. Moreover, fruits with higher TSS are particularly valued by the processing industry [56]. The three-way ANOVA analysis of TSS revealed significant differences arising from the interaction between “WD Treatment” and “Harvest Date” (Figure S3). Regardless of BC treatment, TSS increased by approximately 10%, from the 1st to the 2nd harvest in 0WD plants, confirming previous reports that sugar content typically rises as the harvest season progresses [29]. The two-way ANOVA on °Brix values showed that they remained stable regardless of “WD Treatment” and “BC Treatment” (Table 3). A separate effect of “BC Treatment” and “WD Treatment” was detected during the 2nd harvest (Table 3). Specifically, fruits from BC-treated plants had higher TSS values than those from BC0 plants (Figure 2a), consistent with findings by Sharma et al. [57], who observed a significant enhancement of soluble solids in watermelon following biochar application to the soil. Conversely, TSS levels decreased in fruits harvested from WD-treated plants during the 2nd harvest (Figure 2b). This result contrasts with what was reported in mango and tomato, where WD application was associated with increases in TSS [58,59].

#### 2.5.2. Titratable Acidity (TA) and TSS/TA Ratio

**Figure 3:**
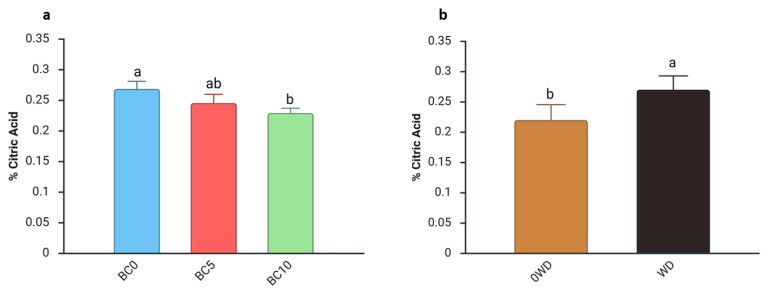
Effect of (**a**) BC Treatment and (**b**) WD Treatment on fruit titratable acidity (% citric acid) in blueberry fruits in the 2nd harvest. Two-way ANOVA, different letters indicate significantly different values at *p* ≤ 0.05 according to Tukey’s test. BC0: 100% Coconut Fiber-CF; BC5: 5% of biochar and 95% of CF (*v*/*v*); BC10: 10% of biochar and 90% of CF (*v*/*v*); 0WD: without wood distillate, WD: treatment with wood distillate.

Acidity, assessed through TA and expressed as % citric acid, is a key determinant of fruit flavor because it reflects both composition and concentration of organic acids [60]. A significant three-way interaction among “BC Treatment”, “WD Treatment”, and “Harvest Date” was detected for TA (Figure S4). Regardless of the BC percentage in the substrate or the WD treatment, fruit acidity generally declined from the first to the second harvest, with an average decrease of 37% (Figure S6). The seasonal reduction in TA aligns with previous studies reporting decreases in acidity accompanied by increases in total soluble solids (TSS) as harvest progresses [61,62,63]. During the 1st harvest, the two-way ANOVA showed an interaction between “BC Treatment” and “WD Treatment” (Table 3). specifically, fruits from BC0 plants treated with WD exhibited a 17% higher TA compared with those grown without WD. During the 2nd harvest, separate effects of the two factors were observed: fruits from BC10-treated plants showed a lower TA than those from BC0 plants (Figure 3a). This decrease in acidity agrees with findings of Ataya et al. [64], who reported reduced fruit acidity in mango fruits following biochar application. Although their study was conducted under soil-based conditions, the similar trend suggests that biochar may influence TA across different cultivation systems. Additionally, an increase in TA was detected in fruits harvested from WD-treated plants during the second harvest (Figure 3b).

In this study, a progressive increase in the TSS/TA ratio was observed as harvest season advanced, with an average rise of 90% from the 1st to the 2nd harvest (Figure S5). In a recent study by Edger et al. [65], the main qualitative parameters defining blueberry fruit at harvest should theoretically fall within the range of >11% TSS, 0.3–1.3% TA, and a TSS/TA ratio between 15 and 30. However, blueberries are typically harvested multiple times within a single season (3–6 harvests, depending on climatic conditions), with hotter climates or longer growing seasons requiring even more frequent harvesting. Such repeated harvests inevitably introduce variability in fruit quality, leading to substantial changes in TSS, TA, and consequently in the TSS/TA ratio. The progressive increase in TSS/TA can be attributed to the asynchronous flowering of blueberry plants, which results in berries at different developmental stages being harvested simultaneously. Early flowers mature first, while later flowers continue to develop, producing berries with lower initial sugar content and higher acidity [66]. For instance, during the second harvest, the TSS/TA ratio ranged between 39.55 and 60.59, mainly due to the increase in sugar concentration observed at this stage of fruit development. The dynamic balance between sweetness and acidity in blueberries is closely linked to changes in TSS and TA during ripening [67]. Consequently, the TSS/TA ratio is widely recognized as a reliable indicator of fruit maturity and a key factor influencing consumer perception [68], with higher values generally associated with more advanced ripening [69], in line with what was found in this study. In the 1st harvest, the two-way ANOVA did not show differences among all treatments performed (Table 3). In contrast, during the second harvest, a significant interaction between “BC Treatment” and “WD Treatment” was observed: fruits from BC0 plants treated with WD showed a 27% decrease in the TSS/TA ratio, while those from BC5 plants exhibited a 14% reduction compared to BC0 plants (Table 3). These results differ from the findings of Abdel-Sattar et al. [58], who reported a significant improvement in the TSS/TA ratio following WD application in mango, suggesting that the effect of WD on this parameter may be species-specific.

#### 2.5.3. Fruit pH

Regarding fruit pH, the three-way ANOVA did not reveal any effect of the “Harvest date”. However, when two-way ANOVAs were performed separately for each harvest, several differences emerged. In both harvest dates, a strong interaction between the two tested factors (“BC Treatment” and “WD Treatment”) was detected (Table 3). Specifically, fruit pH values were consistently lower in all treated plants compared with the BC0 plants that did not receive WD (Table 3). The pH values measured in this study align well with those reported for ripe blueberries in previous research, and are consistent with current market standards [70,71]. Similar to the findings of Li et al. [46], who observed a reduced fruit pH following biochar application in apple orchards, this study also indicate a decrease in fruit pH in response to “BC Treatment”.

### 2.6. Fruit Chemical Characterization

#### 2.6.1. Total Phenolic Content (TPC)

**Figure 4:**
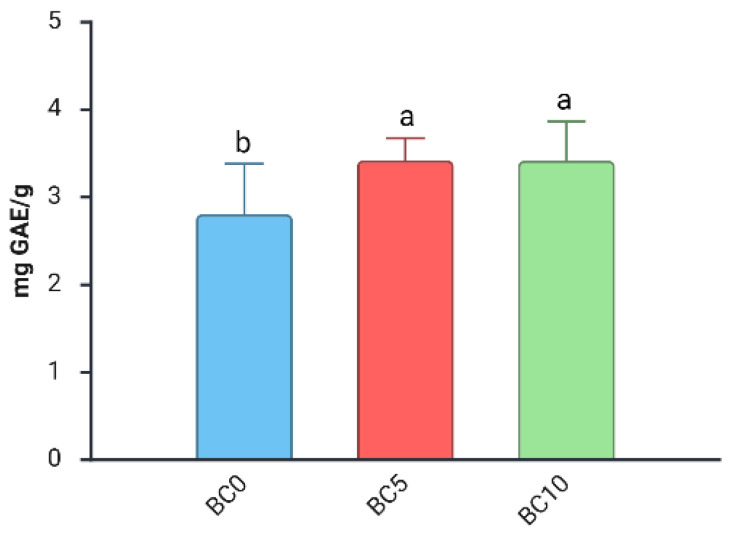
Effect of “BC Treatment” on TPC (Total Polyphenol Content) in blueberry fruits in the 1st harvest. Two-way ANOVA, different letters indicate significantly different values at *p* ≤ 0.05 according to Tukey’s test. BC0: 100% Coconut Fiber-CF; BC5: 5% of biochar and 95% of CF (*v*/*v*); BC10: 10% of biochar and 90% of CF (*v*/*v*).

For TPC, statistical analysis revealed a significant interaction between “BC Treatment,” “WD Treatment,” and “Harvest Date”. A strong effect of “Harvest Date” was observed, with a +57% increase in TPC compared with those from the first, regardless of biochar or WD treatment (Figure S6). This trend is consistent with previous studies reporting higher phenolic concentrations at later harvest stages [29,53]. In the first harvest, only the “BC Treatment” factor had a significant effect on TPC (Table 4). Specifically, fruits from plants grown with BC, regardless of the concentration, had significantly higher total phenolic content compared to those from BC0 plants (Figure 4).

During the second harvest, a significant interaction between the two factors was detected (Table 4), with fruits from BC5 plants treated with WD showing the highest total phenolic content. In particular, BC5WD fruits displayed a 30% increase in TPC compared with fruits from BC5 plants without WD. Notably, the effect of WD emerged only in the second harvest, consistent with findings in tomato [72] and apple [73], where WD application enhanced TPC. However, contrasting results have been reported; Kårlund et al. [74], for example, found no effect of WD on TPC in strawberry, suggesting a possible species-specific responses. It is also noteworthy that the increase in polyphenol content in the present study occurred specifically BC5-treated plants, suggesting a potential synergistic effect between BC, added at this concentration, and WD. This finding is consistent with a previous study on blueberry plants, where a positive trend in polyphenol content was observed following BC and WD treatments, although the differences were not statistically significant [25]. A key distinction of the present study is that the effect became evident at a 5% biochar concentration, whereas Zhang et al. [25] used only 1.5% and 3%, indicating a possible dose-dependent response.

#### 2.6.2. Antioxidant Activity (AO)

Antioxidant activity, measured by the FRAP assay, revealed an interaction among all factors considered in the three-way ANOVA. As illustrated in Figure S7, harvest time had a pronounced effect: overall, AO declined slightly from the first to the second harvest, except in fruits from BC0 plants treated with WD, which displayed a marked increase in AO during the second harvest. Moreover, in the absence of WD, fruits from BC10-treated plants showed no significant differences between harvest dates. During the 1st harvest, an interaction between “BC Treatment” and “WD Treatment” was detected (Table 4). Fruits from BC5 plants receiving WD exhibited higher AO compared to those from BC0 plants under 0WD conditions, while the lowest AO values occurred in fruits from BC0 plants treated with WD. The interaction between the two factors was maintained in the 2nd harvest as well (Table 4). Specifically, AO increased in BC0 fruits following WD application. In contrast, AO decreased in fruits from BC5 and BC10 plants treated with WD relative to their corresponding non-WD controls. Previous studies have highlighted the potential of BC to enhance the nutritional quality of tomato fruits, reporting increases in phenolics, flavonoids, and overall antioxidant activity, depending on the biochar type and application rate [75,76]. Consistent with these findings, the present study also observed an increase in the antioxidant activity of blueberry fruits with BC application. Conversely, WD did not exert a positive effect on AO, in several cases, it even reduced antioxidant activity. This contrasts with the results by Fedeli et al. [59], who observed clear improvements in tomato fruit nutritional quality following WD treatment applied via irrigation or foliar spray. Taken together, these findings underscore the species-specific nature of responses to biostimulant products. It should also be noted that WD often produces stronger effects when applied as a foliar treatment, an approach that was not feasible in the present study.

#### 2.6.3. Anthocyanin Content

**Figure 5:**
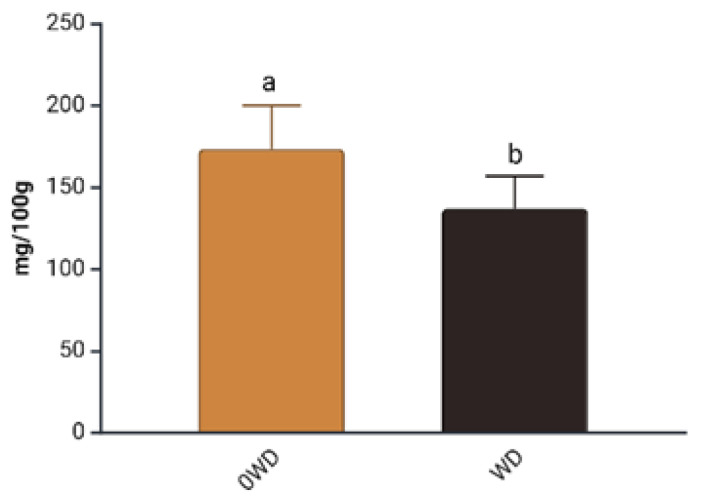
Effect of “WD Treatment” on Anthocyanins content (AC) in blueberry fruits in the 2nd harvest. Two-way ANOVA, different letters indicate significantly different values at *p* ≤ 0.05 according to Tukey’s test. 0WD: without wood distillate, WD: treatment with wood distillate.

Statistical analysis revealed a significant three-way interaction for fruit anthocyanin content (Figure S8). Notably, untreated control plants (BC0, 0WD), showed stable anthocyanin levels between the 1st and 2nd harvests. In contrast, fruits from BC0 plants treated with WD exhibited a marked increase in anthocyanin content in the 2nd harvest (+113%) compared to the 1st. A similar increase between harvests was also observed in fruits from BC5- and BC10-treated plants without WD, with rises of 35% and 78%, respectively. For the corresponding WD-treated plants, however, no statistically significant differences between the harvests were detected (Figure S10). To further explore treatment effects, two-way ANOVAs, were conducted for each harvest date. During the first harvest, a significant interaction was observed (Table 4): anthocyanin content decreased in fruits from BC10 plants compared with BC0 plants when WD was not applied, and a reduction was also detected in BC0 fruits following WD application. Conversely, when WD was applied, BC5 fruits showed higher anthocyanin levels than BC0 fruits. In the 2nd harvest, the two-way ANOVA revealed only a WD effect, with anthocyanin content significantly lower when harvested from plants treated with WD, compared harvested from those not exposed to the WD Treatment (Figure 5). Few studies have investigated the combined effects of BC and WD on berry crops. Zhang et al. [25], one of the few exceptions, reported no significant increase in anthocyanins when applying 1.5% or 3% biochar, alone or incombination with WD, to blueberry plants under field conditions. In contrast, the present study, which employed higher biochar doses, showed a complex and variable influence on anthocyanin levels mirroring its inconsistent effects on antioxidant activita, and its mode of action, likely linked to distribution within the plant, warrants further investigation.

To investigate potential relationships among the measured chemical parameters, Pearson’s correlation analysis was performed. Phenolics and anthocyanins, which are common compounds in blueberries, are commonly expected to correlate positively with antioxidant activity. In this study, a significant positive correlation was found between total phenolic content (TPC) and anthocyanin concentration (*p* = 0.0112), whereas no significant correlations emerged between antioxidant activity (FRAP) and either TPC or anthocyanins (Table 5). These results suggest that other bioactive compounds may contribute to the antioxidant potential in blueberry fruits [77]. Indeed, antioxidant activity in foods depends on their antioxidant content and their chemical structure and both genetics and environmental factors can influence these characteristics [78].

### 2.7. Microbial Community Functional Profile

**Figure 6:**
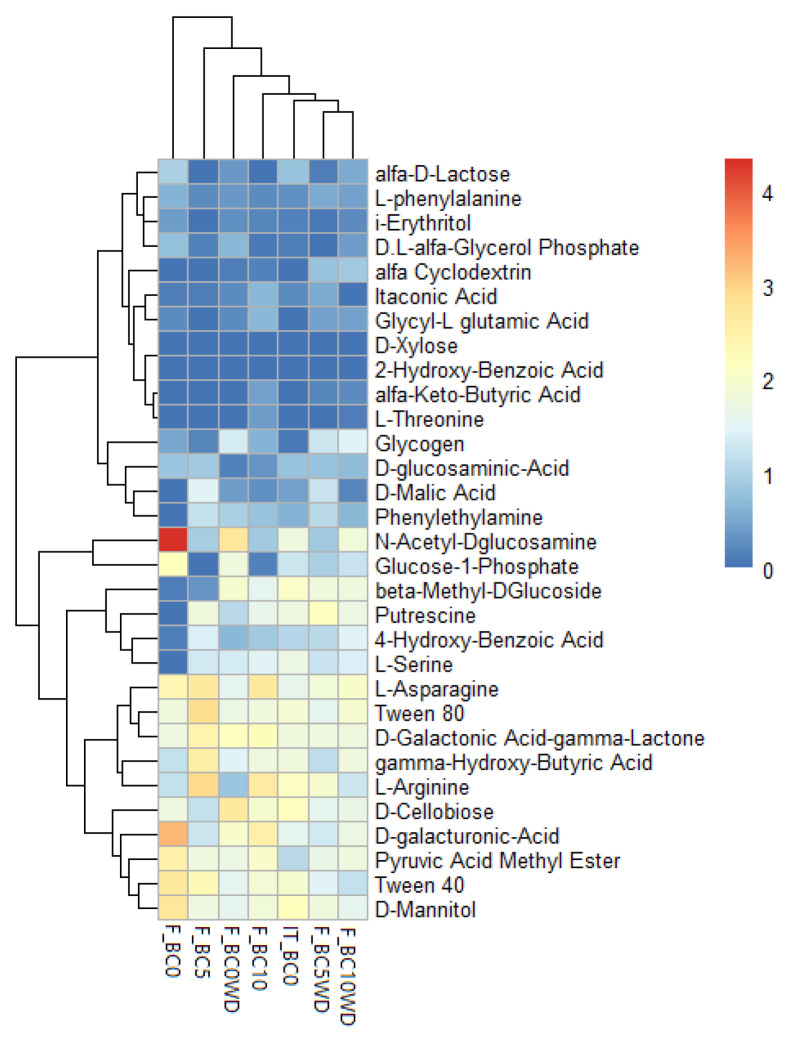
Heatmap of the metabolic profiling of microbial community in different substrate samples (BC0: 100% Coconut Fiber (CF); BC5: 5% of biochar and 95% of CF (*v*/*v*); BC10: 10% of biochar and 90% of CF (*v*/*v*); 0WD: without wood distillate; WD: treatment with wood distillate) compared with BC0 at the initial time (IT_BC0) and at the end of harvesting (F_BC0). Substrate utilization patterns were clustered using Euclidean distance based on normalized Ecoplates data. The color gradient indicates the efficiency of strains in metabolizing the carbon sources.

The Biolog analysis was carried out on the initial substrate sample before treatment (IT_BC0) and after the various treatments at the end of the harvesting season 2024 (F_BC0, F_BC5, F_BC10, F_BC0WD, F_BC5WD; F_BC10WD). Figure 6 presents the results obtained from the Biolog Ecoplate system, displaying a heatmap of the 31 compounds included in the Ecoplate and illustrating which were more or less metabolized by the microbial communities in each sample. Changes are only noticeable in some of the carbon sources. Taking the untreated sample, differences can be seen from the first substrate sampling to the final one. For example, the ability to metabolize N-acetyl-D-glucosamine increased in the final untreated sample (F_BC0) and in some of the treated samples (F_BC5, F_BC0WD, F_BC10WD). Conversely, these final samples did not show ability to metabolize L-arginine compared to the initial sample. During cultivation, changes occurred within the microbial community [79,80]. These observations are supported by the functional indices derived from the Ecoplate data (Table 6).

The AWCD value was significantly lower in the final untreated sample (F_BC0) than in the initial substrate (IT_BC0), which confirms a decline in overall metabolic activity in the absence of treatments. By contrast, all biochar-treated samples, both with and without wood distillate, showed AWCD values that were statistically comparable to those of the initial sample. This indicates that the application of biochar helped to maintain microbial metabolic activity. Substrate richness (SR) followed a similar trend, the lowest value in F_BC0 suggests a loss of functional capacity over the cultivation period, whereas the WD-treated samples displayed the highest richness, reflecting these microbial communities’ ability to metabolise a broader range of substrates. Differences were also observed in Shannon’s diversity index (H): F_BC0 showed a significantly lower value than F_BC10, supporting the idea that higher biochar rates can sustain greater functional diversity.

Moreover, the greater utilization of specific polymers, amino acids, carbohydrates, carboxylic acids, and amines in the treated samples could indicate that the addition of biochar to the substrate promotes the selection of microorganisms specialized in metabolizing these compounds. For instance, the higher utilization of Tween 80 by the microbial community in sample F_BC5, or of putrescine by microorganisms in sample F_BC5WD, supports this observation. This enhanced microbial activity could result in a broader spectrum of essential nutrients available not only to microbes but also to plants [32]. Overall, the Ecoplate data indicate that some shifts in carbon-source utilization occurred over the cultivation period and across the different treatments, suggesting changes in microbial functional activity. Although these patterns should be viewed as indicative, the observations are consistent with general trends reported in the literature regarding the influence of such amendments on microbial communities. It can be concluded that, the shifts in carbon-source utilization detected in this experiment are coherent with previous studies such as Rui et al. [81] and Sivaram et al. [82] reporting that WD applications can alter microbial community structure, increase microbial diversity, and promote the proliferation of taxa specialized in metabolizing easily degradable compounds, ultimately enhancing soil metabolic potential.

## 3. Materials and Methods

### 3.1. Plant Material

The experiment was conducted from February to July 2024, spanning a total of 20 weeks. The trial utilized the Cargo cultivar, a highbush blueberry variety developed through the breeding programs at Fall Creek Farm and Nursery in Lowell, Oregon, USA.

### 3.2. Site Description and Plant Maintenance

The experiment took place at a blueberry-producing farm (I mirtilli di Zeno) located in Ponzano Veneto (TV, Italy) (45.7165, 12.1773). The farm, established in 2021, produces and sells blueberries grown in soilless conditions in 50 L pots (Figure S9). Blueberry plants were maintained under a fertigation system consisting of a mixture of water, acidified water, and a stock solution of fertilizers. The stock solution contained chelated iron, ammonium nitrate, ammonium sulfate, monopotassium phosphate, potassium sulfate, and a blend of micronutrients by Microsol Lampone (composition: B 2%, Cu 0.9%, Mn 17.5%, Mo 0.76%, Zn 5.7%). The fertigation unit delivered the nutrient solution at an electrical conductivity (EC) of 1.3 mS cm^−1^. The final irrigation mixture had a pH of 5.8.

### 3.3. Characteristics of Biochar and Wood Distillate

BC and WD used in this experiment were commercial products purchased from BioDea© Esperia Srl (Arezzo, Italy) [83]. BC is derived from the charring process of materials of plant origin from forestry and agriculture, such as olive pruning residues, marc, bran, fruit kernels, and shells. The properties of the BC (Gold Gravel), as reported by the producer, are detailed in Table 7, while the characteristics of the WD, as reported by the producer, are summarized in Table 8. In addition to assess the WD nutrient content in terms of K^+^, Ca^2+^ and Na^+^, the LAQUAtwin 4M Kit (Horiba Ltd., Kyoto, Japan) was employed, following the manufacturer’s instructions. Briefly, a few drops of WD were applied to the sensor after calibration, and the results were expressed in ppm. All measurements were performed in triplicate.

### 3.4. Experimental Design

The plants used for this experiment had already been in 50 L pots (approximately 42 cm in top diameter × 38 cm in height) for three years, so removing part of the top substrate (about 20 cm) and replacing it with biochar was necessary. This approach was chosen based on literature reports indicating that blueberry plants have a shallow root system [84], which allowed for effective incorporation of the material without disturbing the root architecture. The operation was carried out during the vegetative season, while plants were still in dormancy, to ensure optimal mixing and minimize stress. Although substrate homogeneity was not directly measured, biochar incorporation during this phase, combined with subsequent irrigation cycles during the season, favored progressive mixing and uniform distribution of the material within the upper substrate layer. Each treatment included 15 plants, resulting in a total of 90 plants (6 treatments × 15 plants). The experiment was conducted in the central rows of the commercial soilless blueberry plantation, which hosts several cultivars; only these rows contained the cultivar Cargo, used in the trial. Within this area, plants were assigned to treatments following a randomized block design. A detailed representation of the spatial arrangement and randomization scheme is provided in Figure S10. In addition, plants were arranged following a triangular spacing scheme, with a center-to-center distance of 55 cm between neighboring pots along the oblique axis, and 90 cm between aligned plants in the row, as shown in the scheme (Figure S11). This configuration ensured uniform spacing and provided optimal canopy development space for blueberry plants during the vegetative growth phase.

**Figure 7:**
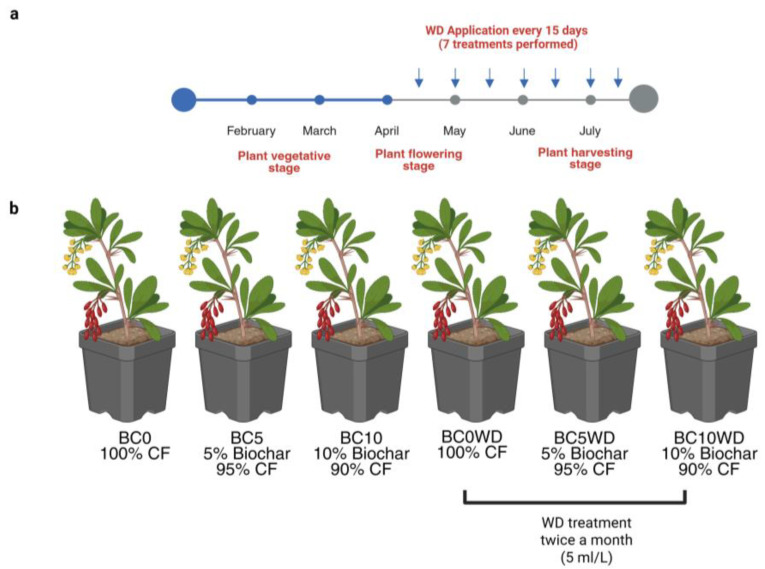
(**a**) Timeline of WD applications during the experimental period. WD was applied twice a month (every 15 days) at a concentration of 5 mL L^−1^, starting from the flowering stage (mid-April) and continuing until harvest (late July), for a total of seven applications. The main phenological stages of the plants (vegetative, flowering, and harvesting) are indicated; (**b**) Representation of the treatments performed in the experimental design. BC0: 100% coconut fiber-CF; BC5: 5% of biochar and 95% of CF (*v*/*v*); BC10: 10% of biochar and 90% of CF (*v*/*v*); BC0WD: BC0 treated with WD; BC5WD: BC5 treated with WD; BC10WD: BC10 treated with WD (Created in BioRender.com. Agosti, A., 2024).

To evaluate the influence of BC incorporation in the substrate and of the WD distribution (2 L/pot at 5 mL L^−1^ at 15-day intervals), as reported in Figure 7a, from the beginning of flowering stage, (mid-April) at 6th week to the harvesting stage (late-July). The experiment consisted of 6 treatments (Figure 7b): (1) BC0: Pots containing 100% coconut fiber; (2) BC5: Pots with a mixture of 5% biochar and 95% coconut fiber (*v*/*v*); (3) BC10: Pots with a mixture of 10% biochar and 90% coconut fiber (*v*/*v*); (4) BC0WD: 100% coconut fiber with WD application; (5) BC5WD: 5% biochar and 95% coconut fiber with WD application; (6) BC10WD: 10% biochar and 90% coconut fiber with WD application.

### 3.5. Plant Growth Measurements

Plant growth measurements were carried out during critical periods of the season. Specifically, the first measurement was taken during plant dormancy (February 2024), two weeks after BC addition in the substrate, the second measurement at flowering (April 2024), and the final measurement at harvest time (July 2024). On these dates, the plant height was measured by considering the three tallest branches of each plant and then calculating an average. Consequently, the growth between each stage was also measured, obtaining the growth rate between the vegetative stage and flowering, as well as between the flowering stage and harvest. This was made possible because, during the February measurement, the selected branches were tagged, and in the subsequent measurements, the same branches were consistently measured.

### 3.6. Substrate pH and EC Monitoring

Substrate pH and electrical conductivity (EC) were manually monitored at three key phenological stages: during the vegetative stage (pH1 and EC1), two weeks after biochar application; at flowering (pH2 and EC2); and at harvest time (pH3 and EC3). Measurements were performed in triplicate directly at the experimental site using a portable pH meter (HI98168—Hanna Instruments—Woonsocket, RI, USA) and a conductivity meter (Hanna Instruments Soil Test™—HI98331).

### 3.7. Physiological Non-Destructive Measurement

Leaf pigment indices, including flavonols (Flv) and SPAD (Soil Plant Analysis Development), were measured non-destructively following the method described by Ali et al. [85]. These measurements were taken from ten fully expanded leaves per plant at the harvest stage in July. A portable multi-pigment meter (MPM-100, ADC BioScientific Ltd., Hoddesdon, UK) was used for data collection. This device determines flavonol content based on fluorescence ratio (F660 nm/F325 nm) while also providing SPAD readings. Additionally, the maximum quantum efficiency of PSII (Fv/Fm) and the leaf fluorescence performance index (PI) were measured before harvest in the morning (8.00 to 11.00 h) using a FluorPen (FP-100, Photon Systems Instruments, Drásov, Czech Republic), following the protocol by Khalid et al. [86]. For these measurements, five fully expanded leaves per plant were selected. Before taking readings, the leaves were dark-adapted for 20 min using light-retaining clips to ensure accurate assessment.

### 3.8. Evaluation of the Plant Production

Fully mature blueberry fruits were collected at two distinct harvesting times, corresponding to mid-July and late July. The yield was determined by measuring the total fruit production per plant throughout the entire harvesting period, providing a basis for evaluating the productivity of each treatment. Measurement was taken in grams (g) using an electronic scale (KERN^®^ EMB 1000-2, Vicenza, Italy). After harvesting, the fruits were stored in a cool box and transferred to the laboratory of the Food and Drug Department, University of Parma, for further analysis. 50 randomly selected fruits from each treatment were used to evaluate the effect of the treatments on the individual fruit weight, caliper, as well as on Total Soluble Solids, treating each fruit as a measurement unit for the purposes of describing fruit size and compositional variability within the treatment. The weight was evaluated with a balance (KERN^®^ EMB 1000-2) and diameter with a manual caliper (Moore & Wright MW110-15DFC Fractional, Fareham, Hampshire, UK).

### 3.9. Characterization of Blueberry Fruits

For the characterization of blueberry fruits, a representative sample was used, collecting 50 fruits per treatment. The parameters, TA, pH, TPC, AO, AC, were measured on whole fruit or blueberry juice, obtained blending blueberry fruits and then passing the juice with Ultra Turrax mixer (IKA^®^, T18 Basic, Oxford, UK) for two 20 s periods, to avoid sample overheating. Chemical parameters were measured on this composite juice sample. All chemical determinations were performed in analytical triplicate (technical replicates of the same pooled sample). As a consequence, the triplicates represent repeat measurements of the same extract rather than biological replication.

#### 3.9.1. Total Soluble Solids Content

Blueberry fruits TSS content, expressed as °Brix, was measured by placing a drop of fruit juice on the lens of an optical portable refractometer (Model Hanna Instruments—Woonsocket, RI, USA).

#### 3.9.2. Titratable Acidity

The TA of the blueberry juice was determined using the titration method [68]. About 5 g of the prepared blueberry juice was taken and diluted with 100 mL of distilled water, phenolphthalein was used as an indicator. TA of blueberry juice was calculated by titrating blueberry juice against 0.1 N NaOH. The acid content of the blueberry fruit sample was calculated based on the volume of NaOH used for neutralizing the acid content in the sample by using the following Equation (1):

(1) $$\%\;citric\;acid=\frac{Vol\;NaOH\left(\mathrm{mL}\right)\times 0.1\left(normality\;NaOH\right)\times 0.064}{g\;Juice}\times 100$$

where 0.064 is the citric acid milliequivalent factor.

Subsequently, the values of TSS and TA were used to calculate the sugar/acid ratio (TSS/TA).

#### 3.9.3. Fruit pH Determination

The pH of the juice was determined by a bench top pH meter (Model LLG-pH meter 5, Hyde Manchester, UK). The instrument was calibrated before each measurement with standard buffer solutions at pH 4.00 and 7.00, and measurements were performed at room temperature (20–22 °C).

#### 3.9.4. Total Phenolic Content

The TPC was determined using the Folin–Ciocalteu phenol reagent [87]. with minor modifications. Briefly, 250 µL of extract—prepared from 0.5 g of blueberry juice with 10 mL of ethanol:water (70:30 *v*/*v*)—was combined with 1 mL of an aqueous solution of Folin–Ciocalteu phenol reagent (Sigma-Aldrich, St. Louis, MO, USA) diluted 1:10 (*v*/*v*) and 2 mL of an aqueous sodium carbonate solution (20%, *w*/*v*). The mixture was incubated in the dark for 30 min, and the absorbance at 760 nm was measured using a spectrophotometer (JASCO V-530, Easton, MD, USA). A calibration curve, based on gallic acid in the concentration range of 10–100 mg/kg (5 points), was used to quantify the polyphenol content in the samples. Each extract was analyzed in duplicate, and the spectrophotometer software was configured to perform three consecutive measurements per sample for improved accuracy. The same procedure was applied to other assays evaluating the antioxidant capacity of the extracts. TPC results were expressed as mg gallic acid equivalents per kilogram of fresh weight (mg GAE/kg FW).

#### 3.9.5. Antioxidant Activity

The antioxidant capacity of the fruits was evaluated by FRAP (Ferric Reducing Antioxidant Power) assay according to Chiancone et al. [88], with absorbance measured at 593 nm using a spectrophotometer (JASCO V-530, Easton, MD, USA). Trolox (Sigma-Aldrich, Stenheim, Germany) solutions (0.1–1 mM) were used to generate the calibration curve. Results, expressed as mM TEAC, were calculated from the absorbance difference between the sample and blank. All solutions were freshly prepared on the day of analysis, and each sample was analyzed in triplicate to ensure accuracy.

#### 3.9.6. Anthocyanin Content

The extraction of anthocyanins was carried out following the method described by Shoebitz et al. [89], with slight modifications. For each treatment, 0.5 g of fresh blueberry fruit was homogenized with 20 mL of methanol/water (50:50, *v*/*v*) acidified with 2 N HCl (final pH 2). The mixture was stirred using a magnetic stirrer for 1 h at room temperature and then centrifuged at 3500 rpm for 15 min. The supernatant was collected, and the residue was re-extracted with 20 mL of acetone/water (70:30, *v*/*v*), stirred again for 60 min, and centrifuged under the same conditions. The two supernatants were combined and used for subsequent analyses. Total anthocyanin content was determined using the differential pH method. Aliquots of 0.3 mL of the extract were mixed separately with 2.7 mL of 0.025 M potassium chloride buffer (pH 1.0) and with 2.7 mL of 0.4 M sodium acetate buffer (pH 4.5). Absorbance was measured at 510 and 700 nm using a JASCO V-530 spectrophotometer (JASCO, Tokyo, Japan). The anthocyanin content was calculated using the molar absorptivity of cyanidin-3-glucoside and expressed as mg cyanidin-3-glucoside equivalents per 100 g of fresh weight (mg·100 g^−1^ FW).

### 3.10. Bacterial Metabolic Profiles

The metabolic capacity of bacterial communities present in the substrate was assessed using Biolog EcoPlates™ (Biolog, Inc., Hayward, CA, USA). This analysis was conducted on soil samples at the end of the treatments. An initial sample taken before the treatment was used as a reference to assess any changes in the microbial community over time. Samples were prepared following the protocol of Nazeer et al. [90] in triplicate. Plates were analysed by the Microplate Reader (dual-wavelength data: OD590) at T0 and after 24, 48 and 72 h to observe the dynamic utilization of different carbon sources from microbes. For kinetic comparative analysis, we take the lecture measurement performed after 48 h (T48) as we observe the maximum change of signal development at this point. The analysis of data was performed using average well color development (AWCD) as a parameter that enables an integral fingerprinting to be captured of the carbon sources used. The value of AWCD was calculated according to Equation (2), as follows:

(2) $$\mathrm{AWCD}\;=\;\frac{\left(C-R\right)}{n}$$

where C is the OD value of each well with a carbon source, R is the OD value of the control well (water), n is the number of wells with carbon sources, and the value of n is 31. We also evaluated the Shannon index (H) resulted from H = −ΣPi ln(Pi), where Pi = ODi/ΣODi, which is the proportional color development of the well over total color development of all wells of a plate. The number of substrates oxidized (substrate richness, SR) was calculated as the sum of the number of cells where ODi value reached 0.15 after 24 h.

### 3.11. Statistical Analysis

The experimental data were analysed using IBM SPSS Statistics version 29.0.1.0 (SPSS Inc., Chicago, IL, USA). Initially, a three-way Analysis of Variance (ANOVA) was performed to evaluate the effects of “BC Treatment” (different percentages of biochar in the substrate), “WD Treatment” (presence or absence of WD), and either “Plant Developmental Stage” or “Harvest Date”, depending on the parameter considered. Nevertheless, because harvest timing exerted a pronounced influence on the majority of the measured variables, separate two-way ANOVAs were performed for each plant developmental stage or harvest date to obtain a more accurate assessment of treatment effects. This analytical approach facilitated a more precise isolation and interpretation of treatment effects under each specific experimental condition. Mean comparisons were carried out using Tukey’s Honestly Significant Difference (HSD) test at a significance level of *p* ≤ 0.05. Statistical analyses of data derived from the Biolog assays were performed in RStudio (version 4.3.2) using the pheatmap package (version 1.0.12) for data visualization and clustering, with default algorithms. To reduce the noise levels, all absorbance values of carbon sources utilisation were referred against the negative control well (A1) and subsequently, all divided by the respective AWCD. Negative values were set to 0. Normalized data were used for statistical analysis using one-way ANOVA. Mean comparisons were carried out using Tukey’s Honestly Significant Difference (HSD) test at a significant level of *p* ≤ 0.05.

## 4. Conclusions

This study provides valuable insights into the effects of biochar and wood distillate on the growth, yield, and fruit quality of blueberry plants cultivated in a soilless system. Among the tested treatments, the incorporation of 10% biochar into the substrate proved to be the most effective strategy, leading to the highest fruit yield without compromising plant growth or quality parameters. This improvement could be related to the enhanced substrate porosity, nutrient availability, and microbial activity associated with the integration of biochar in the growing substrate. Indeed, the Biolog analysis indicated that the addition of biochar and wood distillate can modulate microbial functional profiles, leading to measurable shifts in carbon-source utilization. Conversely, wood distillate alone or in combination with the biochar did not have a positive influence on many of the tested parameters. Nonetheless, 5% BC combined with WD promoted higher total polyphenol content, indicating a potential synergistic effect in improving fruit nutraceutical quality. From a practical standpoint, these results indicate that a moderate biochar incorporation (5–10%) in the growing substrate can be recommended as a sustainable practice to reduce the use of less sustainable substrate in soilless blueberry cultivation, ensuring, at the same time, an improvement of yield and fruit quality. The application of wood distillate, however, may require further optimization in terms of concentration and timing. Because this study reflects the first year of cultivation after biochar application, long-term trials are needed to determine whether the observed benefits of biochar on plants persist, in particular with respect to key physicochemical properties such as substrate pH stability. These considerations highlight the need for future research focused on (i) evaluating multi-year responses to biochar, (ii) optimizing wood distillate concentration and application frequency, and (iii) investigating substrate dynamics to better understand the long-term sustainability of the treatments carried out. Overall, the present study supports the integration of biochar as a key component in sustainable soilless blueberry production systems, and contributes to circular economy practices through the valorization of biomass-derived materials.

## Figures and Tables

**Table 1:** Effect of “BC Treatment” and “WD Treatment” on pH and Electrical Conductivity (EC) of the substrate in three different plant developmental stages: flowering and harvesting.

BC Treatment	WD Treatment	pH1	pH2	pH3	EC1	EC2	EC3
					mS cm^−1^	mS cm^−1^	mS cm^−1^
BC0	0WD	5.3 ± 0.2	4.7 ± 0.2	5.5 ± 0.1 ^b^	0.07 ± 0.001 ^bc^	0.09 ± 0.003 ^b^	0.16 ± 0.03
BC5		6.2 ± 0.2	6.9 ± 0.1	5.4 ± 0.1 ^b^	0.074 ± 0.007 ^bc^	0.09 ± 0.02 ^b^	0.22 ± 0.02
BC10		6.6 ± 0.2	7.0 ± 0.1	6.0 ± 0.1 ^a^	0.102 ± 0.007 ^ab^	0.104 ± 0.009 ^b^	0.21 ± 0.02
BC0	WD	6.1 ± 0.1	4.7 ± 0.1	4.6 ± 0.1 ^c^	0.056 ± 0.004 ^c^	0.1 ± 0.01 ^b^	0.159 ± 0.001
BC5		7.14 ± 0.05	7.0 ± 0.3	5.8 ± 0.04 ^ab^	0.12 ± 0.02 ^a^	0.13 ± 0.02 ^b^	0.19 ± 0.01
BC10		7.2 ± 0.1	7.1 ± 0.1	5.6 ± 0.1 ^ab^	0.138 ± 0.005 ^a^	0.25 ± 0.01 ^a^	0.202 ± 0.008
Statistical analysis of the factors							
BC Treatment (BC)		<0.001	<0.001	<0.001	<0.001	<0.001	0.051
WD Treatment (WD)		<0.001	0.806	0.003	0.013	<0.001	0.446
BC × WD		0.675	0.954	<0.001	0.016	<0.001	0.821

Two-way ANOVA at different plant developmental stages (identified with 1, 2 or 3): early flowering (1), late flowering (2) and harvesting (3) followed by Tukey’s post hoc test (*p* ≤ 0.05) for normally distributed data. Results are expressed as mean ± standard error. Within each parameter and within each plant developmental stage, different letters indicate statistically different values. Abbreviations: BC0: 100% Coconut Fiber-CF; BC5: 5% of biochar and 95% of CF (*v*/*v*); BC10: 10% of biochar and 90% of CF (*v*/*v*); 0WD: without wood distillate, WD: treatment with wood distillate, EC: Electrical Conductivity.

**Table 2:** Influence of “BC Treatment” and “WD Treatment” on blueberry fruit production.

BC Treatment	WD Treatment	Yield Plant^−1^ (g)
BC0	0WD	925 ± 22 ^cd^
BC5		1169 ± 44 ^b^
BC10		1350 ± 22 ^a^
BC0	WD	1006 ± 3 ^bc^
BC5		830 ± 29 ^e^
BC10		1094 ± 23 ^ab^
Statistical analysis of the factors		
BC Treatment (BC)		<0.001
WD Treatment (WD)		<0.001
BC × WD		<0.001

Two-way ANOVA followed by Tukey’s post hoc test (*p* ≤ 0.05). Results are expressed as mean ± standard error. Within each parameter and different letters indicate statistically different values. Abbreviations: BC0: 100% Coconut Fiber-CF; BC5: 5% of biochar and 95% of CF (*v*/*v*); BC10: 10% of biochar and 90% of CF (*v*/*v*); 0WD: without wood distillate, WD: treatment with wood distillate.

**Table 3:** Influence of “BC Treatment” and “WD Treatment” on blueberry fruits weight, caliper, TSS, TA, TSS/TA, and pH on different harvest dates.

Harvest Date	BC Treatment	WD Treatment	Weight (g)	Caliper (mm)	TSS (°Brix)	TA (%Citric Acid)	TSS/TA	pH
1st harvest	BC0	0WD	2.18 ± 0.08	17.8 ± 0.2	11.8 ± 0.5	0.37 ± 0.02 ^b^	28 ± 1	3.17 ± 0.02 ^a^
	BC5		2.42 ± 0.09	18.5 ± 0.2	12.9 ± 0.2	0.38 ± 0.01 ^b^	28.9 ± 0.7	2.99 ± 0.01 ^b^
	BC10		2.3 ± 0.09	17.8 ± 0.2	12.7 ± 0.3	0.4 ± 0.01 ^ab^	27 ± 1	2.90 ± 0.01 ^c^
	BC0	WD	2.4 ± 0.1	18.5 ± 0.3	10.9 ± 0.4	0.43 ± 0.01 ^a^	26 ± 1	2.98 ± 0.03 ^b^
	BC5		2.34 ± 0.09	18.0 ± 0.2	11.8 ± 0.4	0.39 ± 0.01 ^b^	28 ± 2	2.94 ± 0.01 ^bc^
	BC10		2.23 ± 0.07	18.1 ± 0.2	12.7 ± 0.3	0.4 ± 0.01 ^b^	28 ± 1	2.89 ± 0.01 ^c^
Statistical analysis of the factors								
BC Treatment			0.436	0.462	0.144	0.243	0.473	<0.001
WD Treatment			0.673	0.368	0.914	0.006	0.998	<0.001
BC × WD			0.134	0.070	0.701	0.001	0.385	<0.001
2nd harvest	BC0	0WD	1.47 ± 0.08 ^abc^	15.9 ± 0.2 ^abc^	11.3 ± 0.7	0.25 ± 0.02	54.7 ± 0.7 ^a^	2.99 ± 0.05 ^a^
	BC5		1.84 ± 0.06 ^bc^	16.7 ± 0.2 ^c^	11.1 ± 0.7	0.21 ± 0.01	60 ± 2 ^a^	2.98 ± 0.01 ^b^
	BC10		1.68 ± 0.06 ^a^	16.2 ± 0.2 ^a^	11.4 ± 0.5	0.22 ± 0.01	60 ± 1 ^a^	2.91 ± 0.02 ^b^
	BC0	WD	1.72 ± 0.07 ^c^	16.5 ± 0.2 ^bc^	10.4 ± 0.3	0.28 ± 0.01	39 ± 2 ^c^	3.18 ± 0.04 ^b^
	BC5		1.56 ± 0.05 ^ab^	15.8 ± 0.2 ^ab^	11.0 ± 0.3	0.27 ± 0.01	47 ± 1 ^b^	2.95 ± 0.02 ^b^
	BC10		1.9 ± 0.1 ^abc^	17.0 ± 0.3 ^abc^	10.6 ± 0.5	0.24 ± 0.01	56 ± 2 ^a^	2.95 ± 0.03 ^b^
Statistical analysis of the factors								
BC Treatment			0.026	0.137	<0.001	0.014	<0.001	<0.001
WD Treatment			0.268	0.424	0.026	0.001	<0.001	<0.001
BC × WD			<0.001	<0.001	0.315	0.264	0.002	<0.001

Two-way ANOVA for each harvest date, considering the factors “Treatment” and “WD Presence”. Tukey’s post hoc test (*p* ≤ 0.05). Within each parameter and within each harvest date, different letters indicate statistically different values. Results are expressed as mean ± standard error. 1st harvest: early July, 2nd harvest: late July. Abbreviations: BC0: 100% Coconut Fiber-CF; BC5: 5% of biochar and 95% of CF (*v*/*v*); BC10: 10% of biochar and 90% of CF (*v*/*v*); WD: treatment with wood distillate; 0WD: without wood distillate. TSS: Total Soluble Solids; TA: Titratable acidity.

**Table 4:** “BC Treatment”, and “WD Treatment” on fruit chemical parameters (TPC, FRAP, and AC) on different harvest dates.

Harvest Date	BC Treatment	WD Treatment	TPC (mg GAE/g)	FRAP (mM TEAC)	AC (mg/100 g)
1st harvest	BC0	0WD	2.5 ± 0.3	4.8 ± 0.1 ^bc^	134 ± 16 ^ab^
	BC5		3.4 ± 0.2	5.41 ± 0.09 ^b^	145 ± 13 ^a^
	BC10		3.4 ± 0.2	5.00 ± 0.02 ^ab^	95 ± 9 ^bc^
	BC0	WD	3.0 ± 0.2	4.65 ± 0.15 ^b^	86 ± 6 ^c^
	BC5		3.41 ± 0.06	5.41 ± 0.02 ^a^	134 ± 2 ^ab^
	BC10		3.4 ± 0.3	5.01 ± 0.04 ^ab^	114 ± 2 ^abc^
Statistical analysis of the factors					
BC Treatment			0.020	<0.001	0.009
WD Treatment			0.391	0.233	0.134
BC Treatment × WD Treatment			0.406	0.004	0.017
2nd harvest	BC0	0WD	4.47 ± 0.01 ^b^	4.63 ± 0.02 ^b^	156 ± 7
	BC5		4.7 ± 0.1 ^b^	5.1 ± 0.1 ^a^	196 ± 19
	BC10		5.4 ± 0.4 ^ab^	5.3 ± 0.1 ^a^	169 ± 10
	BC0	WD	4.75 ± 0.15 ^b^	5.04 ± 0.02 ^a^	152 ± 9
	BC5		6.1 ± 0.3 ^a^	4.30 ± 0.03 ^b^	131 ± 15
	BC10		4.9 ± 0.1 ^b^	4.6 ± 0.1 ^b^	12 ± 6
Statistical analysis of the factors					
BC Treatment (BC)			0.008	0.105	0.485
WD Treatment (WD)			0.055	<0.001	0.003
BC × WD			0.002	<0.001	0.485

Two-way ANOVA for each harvest date, considering the factors “BC Treatment” and “WD Treatment”. Tukey’s post hoc test (*p* ≤ 0.05). Within each parameter and within each harvest date, different letters indicate statistically different values. Results are expressed as mean ± standard error. 1st harvest: early July, 2nd harvest: late July. BC0: 100% Coconut Fiber-CF; BC5: 5% of biochar and 95% of CF (*v*/*v*); BC10: 10% of biochar and 90% of CF (*v*/*v*); WD: treatment with wood distillate; 0WD: without wood distillate. TPC: Total Polyphenol Content; AO: Antioxidant Activity; AC: Anthocyanin content.

**Table 5:** Correlation matrix among chemical parameters in blueberry samples.

	FRAP	TPC	AC
FRAP	1	−0.28338	0.240424
TPC		1	**0.417883**
AC			1

Pearson’s correlation matrix between antioxidant activity (FRAP), total phenolic content (TPC), and anthocyanin content (AC) in blueberry samples. Correlation coefficients were calculated using the Pearson correlation analysis. Significant correlations are indicated in bold (*p* ≤ 0.05).

**Table 6:** Metabolic functional diversity of microorganisms in different samples.

Index	IT_BC0	F_BC0	F_BC5	F_BC10	F_BC5WD	F_BC10WD
AWCD	0.9 ± 0.01 ^a^	0.3 ± 0.01 ^b^	0.6 ± 0.01 ^ab^	0.8 ± 0.06 ^a^	0.9 ± 0.01 ^a^	0.9 ± 0.05 ^a^
SR	16 ± 0.1 ^ab^	10 ± 0.1 ^b^	14.5 ± 0.7 ^ab^	13.5 ± 0.7 ^ab^	17 ± 0.1 ^a^	16 ± 0.01 ^ab^
H	3 ± 0.1 ^ab^	2.7 ± 0.3 ^b^	2.9 ± 0.01 ^ab^	3.1 ± 0.01 ^a^	3 ± 0.01 ^ab^	3.2 ± 0.04 ^a^

One-way ANOVA, Tukey’s test, *p* ≤ 0.05. Within each parameter different letters indicate values statistically different. Abbreviations: AWCD: average well color development; SR: substrate richness; H: Shannon index.

**Table 7:** Physico-chemical characteristics of biochar used in this study [83].

Particle Diameter (µm)	<500, <2, <5
Nitrogen (%)	<0.5
Potassium (g Kg^−1^)	3.020
Phosphorous (%)	0.340
Calcium (g Kg^−1^)	9.920
Magnesium (g Kg^−1^)	0.852
Sodium (g Kg^−1^)	0.291
Total moisture (%)	>10
Carbon (%)	70
Water holding capacity (Max, %)	80
Salinity (mS cm^−1^)	1.1
pH	9.85
Ash content (%)	4.6
Molar H/C ratio	0.2

**Table 8:** Characteristics of wood distillate (BioDea^®^), as indicated by the producer (Bio-Esperia srl) [83].

pH	4.00 ± 0.5
Density	1.05 Kg L^−1^
Acetic acid	2.1 ± 0.1 (% *v*/*v*)
Total phenolic Content	3.0 ± 0.2 g Kg^−1^
Total Polyphenol Content	24.00 ± 2.00 g Kg^−1^
Heavy metals content	<1 mg Kg^−1^
K^+^	22 ± 1 ppm
Ca^2+^	318 ± 13 ppm
Na^+^	98 ± 11 ppm

## Data Availability

All data necessary to support the findings of this study are presented within the article and its Supplementary Materials. Additional raw measurements that are not essential for reproducing the analyses can be provided by the corresponding author upon reasonable request.

## Supplementary Materials

The following supporting information can be downloaded at: https://www.mdpi.com/article/10.3390/plants14243773/s1, Table S1: Effect of the “BC Treatment” and the “WD Treatment” on Performance Index (PI), Fv/Fm, Flv and SPAD at harvest time; Figure S1: Effect of “BC Treatment”, “WD Treatment” and “Harvest date” on blueberry fruit weight; Figure S2: Effect of “BC Treatment”, “WD Treatment” and “Harvest date” on blueberry fruit caliper; Figure S3: Effect of WD Presence and Harvest date on blueberry fruit Total Soluble Solids; Figure S4: Effect of “BC Treatment”, “WD Treatment” and “Harvest date” on blueberry fruit acidity expressed as % citric acid; Figure S5: Effect of “BC Treatment” and “Harvest date” on blueberry fruit TSS/TA; Figure S6: Effect of “BC Treatment”, “WD Treatment” and “Harvest date” on blueberry fruit Total Phenolic Content content; Figure S7: Effect of “BC Treatment”, “WD Treatment” and “Harvest date” on blueberry fruit Antioxidant Activity expressed as mM TEAC; Figure S8: Effect of “BC Treatment”, “WD Treatment” and “Harvest date” on blueberry fruit Anthocyanins content expressed as mg/100 g; Figure S9: Experimental site at “I mirtilli di Zeno” farm (Farm SABIBI), in Ponzano Veneto, Treviso, Italy; Figure S10: Spatial layout of the experimental area and randomized block design; Figure S11: Triangular spacing scheme used in the experimental design for blueberry plants.

## References

[1] FAOSTAT (2025). https://www.fao.org/faostat/en/#home.

[2] Brondino L., Borra D., Giuggioli N.R., Massaglia S. (2021). Mechanized blueberry harvesting: Preliminary results in the Italian context. Agriculture.

[3] Cordes H., Iriarte A., Villalobos P. (2016). Evaluating the carbon footprint of chilean organic blueberry production. Int. J. Life Cycle Assess..

[4] Fang Y., Nunez G., Fisher P., Munoz P.R. (2022). Effect of container size, substrate composition, and genotype on growth and fruit quality of young southern highbush blueberry in a container-based intensive production system. Sci. Hortic..

[5] Gilbert J.L., Olmstead J.W., Colquhoun T.A., Levin L.A., Clark D.G., Moskowitz H.R. (2014). Consumer-assisted selection of blueberry fruit quality traits. HortScience.

[6] Almutairi K.F., Machado R.M., Bryla D.R., Strik B.C. (2017). Chemigation with micronized sulfur rapidly reduces soil pH in a new planting of northern highbush blueberry. HortScience.

[7] Caspersen S., Svensson B., Håkansson T., Winter C., Khalil S., Asp H. (2016). Blueberry—Soil interactions from an organic perspective. Sci. Hortic..

[8] Salazar-Gutiérrez M.R., Lawrence K., Coneva E.D., Chaves-Córdoba B. (2023). Photosynthetic Response of Blueberries Grown in Containers. Plants.

[9] Smrke T., Veberic R., Hudina M., Zitko V., Ferlan M., Jakopic J. (2021). Fruit Quality and Yield of Three Highbush Blueberry (*Vaccinium corymbosum* L.) Cultivars Grown in Two Planting Systems under Different Protected Environments. Horticulturae.

[10] Li T., Bi G. (2019). Container production of southern highbush blueberries using high tunnels. HortScience.

[11] Heller C.R., Nunez G.H. (2022). Preplant fertilization increases substrate microbial respiration but does not affect southern highbush blueberry establishment in a coconut coir-based substrate. HortScience.

[12] Lemaire F. (1995). Physical, chemical and biological properties of growing medium. Acta Hortic..

[13] Carlile W.R., Cattivello C., Zaccheo P. (2015). Organic growing media: Constituents and properties. Vadose Zone J..

[14] Awang Y., Shaharom A.S., Mohamad R.B., Selamat A. (2009). Chemical and physical characteristics of cocopeat-based media mixtures and their effects on the growth and development of Celosia cristata. Am. J. Agric. Biol. Sci..

[15] Gruda N.S. (2019). Increasing sustainability of growing media constituents and stand-alone substrates in soilless culture systems. Agronomy.

[16] Kaushal A., Yadav R.K., Singh N. (2024). Biochar application in sustainable production of horticultural crops in the new era of soilless cultivation. Biochar Production for Green Economy.

[17] Grewal A., Abbey L., Gunupuru L.R. (2018). Production, prospects and potential application of pyroligneous acid in agriculture. J. Anal. Appl. Pyrol..

[18] Kang M.W., Yibeltal M., Kim Y.H., Oh S.J., Lee J.C., Kwon E.E., Lee S.S. (2022). Enhancement of soil physical properties and soil water retention with biochar-based soil amendments. Sci. Total Environ..

[19] Guo L., Yu H., Kharbach M., Zhang W., Wang J., Niu W. (2021). Biochar improves soil-tomato plant, tomato production, and economic benefits under reduced nitrogen application in northwestern China. Plants.

[20] Sales B.K., Bryla D.R., Trippe K.M., Scagel C.F., Strik B.C., Sullivan D.M. (2022). Biochar as an Alternative Soil Amendment for Establishment of Northern Highbush Blueberry. HortScience.

[21] Lehmann J., Joseph S. (2009). Biochar for Environmental Management: Science, Technology and Implementation.

[22] Valero D., Serrano M. (2010). Postharvest Biology and Technology for Preserving Fruit Quality.

[23] Shang X., Hung C.Y., Husk B., Orsat V., Whalen J.K. (2021). Wood-based biochar for small fruit production in southern Quebec, Canada. Can. J. Soil Sci..

[24] Ortiz-Delvasto N., Garcia-Ibañez P., Olmos-Ruiz R., Bárzana G., Carvajal M. (2023). Substrate composition affects growth and physiological parameters of blueberry. Sci. Hortic..

[25] Zhang Y., Wang X., Liu B., Liu Q., Zheng H., You X., Sun K., Luo X., Li F. (2020). Comparative study of individual and Co-Application of biochar and wood vinegar on blueberry fruit yield and nutritional quality. Chemosphere.

[26] Souza J.B.G., Ré-Poppi N., Raposo J.L. (2012). Characterization of pyroligneous acid used in agriculture by gas chromatography-mass spectrometry. J. Braz. Chem. Soc..

[27] Guo W., Nazim H., Liang Z., Yang D. (2016). Magnesium deficiency in plants: An urgent problem. Crop J..

[28] Becagli M., Santin M., Cardelli R. (2022). Co-application of wood distillate and biochar improves soil quality and plant growth in basil (*Ocimum basilicum*). J. Plant Nutr. Soil Sci..

[29] Zydlik Z., Zydlik P., Kafkas N.E., Yesil B., Cieśliński S. (2022). Foliar application of some macronutrients and micronutrients improves yield and fruit quality of highbush blueberry (*Vaccinium corymbosum* L.). Horticulturae.

[30] Zorenc Z., Veberic R., Stampar F., Koron D., Mikulic-Petkovsek M. (2016). Changes in berry quality of northern highbush blueberry (*Vaccinium corymbosum* L.) during the harvest season. Turk. J. Agric..

[31] Godara A., Ames Z.R., Deltsidis A. (2025). Delayed Harvest Reduces Postharvest Quality and Storability of Southern Highbush cv. Meadowlark and Rabbiteye Blueberry cv. Brightwell. HortScience.

[32] Agosti A., Nazeer S., Del Vecchio L., Leto L., Di Fazio A., Hadj-Saadoun J., Levante A., Rinaldi M., Dhenge R., Lazzi C. (2024). Effect of Biochar and Wood Distillate on Vegeto-Productive Performances of Tomato (*Solanum lycopersicum* L.) Plants, var. Solarino, Grown in Soilless Conditions. Agronomy.

[33] Fedeli R., Cruz C., Loppi S., Munzi S. (2024). Hormetic Effect of Wood Distillate on Hydroponically Grown Lettuce. Plants.

[34] Ramaroson M.L., Koutouan C., Helesbeux J.J., Le Clerc V., Hamama L., Geoffriau E., Briard M. (2022). Role of phenylpropanoids and flavonoids in plant resistance to pests and diseases. Molecules.

[35] Islam M.R., Haque K.S., Akter N., Karim M.A. (2014). Leaf chlorophyll dynamics in wheat based on SPAD meter reading and its relationship with grain yield. Sci. Agric..

[36] Xu J., Meng J. (2016). Research progress and prospect of remote sensing estimation of crop chlorophyll content. Remote Sens. Technol. Appl..

[37] Chrysargyris A., Prasad M., Tzortzakis N. (2024). Wood-Based biochar ratio used for partial Peat replacement ingrowing media for *Antirrhinum majus* pot production. Agriculture.

[38] Ren T., Wang H., Yuan Y., Feng H., Wang B., Kuang G., Wei Y., Gao W., Shi H., Liu G. (2021). Biochar increases tobacco yield by promoting root growth based on a three-year field application. Sci. Rep..

[39] Hafez E.M., Gao Y., Alharbi K., Chen W., Elhawat N., Alshaal T., Osman H.S. (2024). Antioxidative and Metabolic Responses in Canola: Strategies with Wood Distillate and Sugarcane Bagasse Ash for Improved Growth under Abiotic Stress. Plants.

[40] Wang S., Zheng J., Wang Y., Yang Q., Chen T., Chen Y., Chi D., Xia G., Siddique K.H.M., Wang T. (2021). Photosynthesis, chlorophyll fluorescence, and yield of peanut in response to biochar application. Front. Plant Sci..

[41] Li Z.X., Li R.J., Mu J., Yang Z.L., Sun S., Yan Y., Wang G.Y. (2020). Effects of biochar on physiological characteristics of cucumber seedlings in diethyl hexyl phthalate contaminated soil. Plant Physiol. J..

[42] Liu X., Zhang J., Wang Q., Chang T., Shaghaleh H., Hamoud Y.A. (2022). Improvement of photosynthesis by biochar and vermicompost to enhance tomato (*Solanum lycopersicum* L.) yield under greenhouse conditions. Plants.

[43] Ali A., Santoro P., Ferrante A., Cocetta G. (2023). Investigating pulsed LED effectiveness as an alternative to continuous LED through morpho-physiological evaluation of baby leaf lettuce (*Lactuca sativa* L. var. Acephala). S. Afr. J..

[44] Fedeli R., Loppi S., Cruz C., Munzi S. (2024). Evaluating Seawater and Wood Distillate for Sustainable Hydroponic Cultivation: Implications for Crop Growth and Nutritional Quality. Sustainability.

[45] Retamales J.B., Hancock J.F. (2018). Blueberries.

[46] Li W., Gao J., Zhou S., Zhou F. (2024). Effect of Biochar on Apple Yield and Quality in Aged Apple Orchards on the Loess Plateau (China). Agronomy.

[47] Vaccari F.P.A., Maienza F., Miglietta S., Baronti S., Di Lonardo L., Giagnoni A., Lagomarsino A., Pozzi E., Pusceddu E., Ranieri R. (2015). Biochar stimulates plant growth but no fruit yield of processing tomato in a fertile soil. Agr. Ecosyst. Environ..

[48] Zulkarami B., Ashrafuzzaman M., Husni M.O., Ismail M.R. (2011). Effect of pyroligneous acid on growth, yield and quality improvement of rockmelon in soilless culture. Aust. J. Crop Sci..

[49] Castrejón A.D.R., Eichholz I., Rohn S., Kroh L.W., Huyskens-Keil S. (2008). Phenolic profile and antioxidant activity of highbush blueberry (*Vaccinium corymbosum* L.) during fruit maturation and ripening. Food Chem..

[50] Iqbal J., Kiran S., Hussain S., Iqbal R.K., Ghafoor U., Younis U., Zarei T., Naz M., Germi S.G., Danish S. (2021). Acidified biochar confers improvement in quality and yield attributes of sufaid chaunsa mango in saline soil. Horticulturae.

[51] Mungkunkamchao T., Kesmala T., Pimratch S., Toomsan B., Jothityangkoon D. (2013). Wood vinegar and fermented bioextracts: Natural products to enhance growth and yield of tomato (*Solanum lycopersicum* L.). Sci. Hortic..

[52] Connor A.M., Luby J.J., Tong C.B.S. (2002). Variability in antioxidant activity in blueberry and correlations among different antioxidant activity assays. J. Am. Hort. Soc..

[53] Mallik A.U., Hamilton J. (2017). Harvest date and storage effect on fruit size, phenolic content and antioxidant capacity of wild blueberries of NW Ontario, Canada. J. Food Sci. Technol..

[54] Prior R.L., Cao G., Martin A., Sofic E., McEwen J., O’Brien C., Lischner N., Ehlenfeldt M., Kalt W., Krewer G. (1998). Antioxidant capacity as influenced by total phenolic and anthocyanin content, maturity, and variety of *Vaccinium* species. J. Agric. Food Chem..

[55] Echeverría G., Cantín C.M., Ortiz A., López M.L., Lara I., Graell J. (2015). The impact of maturity, storage temperature and storage duration on sensory quality and consumer satisfaction of ‘Big Top^®^’nectarines. Sci. Hortic..

[56] Stajčić M.S., Tepić N.A., Djilas M.S., Šumić M.Z., Čanadanović-Brunet M.J., Ćetković G., Vulić J., Šaponjac V.T. (2012). Chemical composition and antioxidant activity of berry fruit. Acta Period. Technol..

[57] Sharma G., Banik D., Mehta C.M., Eiji N., Inubushi K. (2025). Influence of Biochar Application Rates on Watermelon Growth, Yield, and Soil Nutrient Availability. J. Food Chem. Nanotechnol..

[58] Abdel-Sattar M., Mostafa L.Y., Rihan H.Z. (2024). Enhancing mango productivity with wood vinegar, humic acid, and seaweed extract applications as an environmentally friendly strategy. Sustainability.

[59] Fedeli L., Marotta L., Frattaruolo A., Panti G., Carullo F., Fusi S., Saponara S., Gemma Butini S., Cappello A.R., Vannini A. (2023). Nutritionally enriched tomatoes (*Solanum lycopersicum* L.) grown with wood distillate: Chemical and biological characterization for quality assessment. J. Food Sci..

[60] Xu K., Wang A., Brown S. (2012). Genetic characterization of the Ma locus with pH and titratable acidity in apple. Mol. Breed..

[61] Gibson L., Rupasinghe H., Forney C., Eaton L. (2013). Characterization of changes in polyphenols, antioxidant capacity and physico-chemical parameters during lowbush blueberry fruit ripening. Antioxidants.

[62] Lin Y., Huang G., Zhang Q., Wang Y., Dia V.P., Meng X. (2020). Ripening affects the physicochemical properties, phytochemicals and antioxidant capacities of two blueberry cultivars. Postharvest Biol. Technol..

[63] Ribera-Fonseca A., Noferini M., Rombola A.D. (2016). Non-destructive assessment of highbush blueberry fruit maturity parameters and anthocyanins by using a visible/ near infrared (vis/nir) spectroscopy device: A preliminary approach. J. Soil Sci. Plant Nutr..

[64] Ataya S.M., Mansour N., Saudy H.S. (2025). Combined Effects of Biochar and 2–Hydroxybenzoic Acid on Ameliorating the Nutritional Status, Productivity and Fruit Quality of Salt Stress-Imposed Mango Trees. J. Soil Sci. Plant Nutr..

[65] Edger P.P., Iorizzo M., Bassil N.V., Benevenuto J., Ferrão L.F.V., Giongo L., Zalapa J. (2022). There and back again; historical perspective and future directions for Vaccinium breeding and research studies. Hortic. Res..

[66] Gasdick M., Dick D., Mayhew E., Lobos G., Moggia C., VanderWeide J. (2025). First they’re sour, then they’re sweet: Exploring the berry-to-berry uniformity of blueberry quality at harvest and implications for consumer liking. Postharvest Biol. Technol..

[67] De Vetter L.W., Yang W.Q., Takeda F., Korthuis S., Li C. (2019). Modified over-therow machine harvesters to improve northern highbush blueberry fresh fruit quality. Agriculture.

[68] Rivera S., Kerckhoffs H., Sofkova-Bobcheva S., Hutchins D., East A. (2021). Influence of water loss on mechanical properties of stored blueberries. Postharvest Biol. Technol..

[69] Moggia C., Graell J., Lara I., González G., Lobos G.A. (2017). Firmness at harvest impacts postharvest fruit softening and internal browning development in mechanically damaged and non-damaged highbush blueberries (*Vaccinium corymbosum* L.). Front. Plant Sci..

[70] Aliman J., Michalak I., Busatlic E., Aliman L., Kulina M., Radovic M., Hasanbegovic J. (2020). Study of the physicochemical properties of highbush blueberry and wild bilberry fruit incentral Bosnia. Turk. J. Agric..

[71] Hwang H., Kim Y.J., Shin Y. (2020). Assessment of physicochemical quality, antioxidant content and activity, and inhibition of cholinesterase between unripe and ripe blueberry fruit. Foods.

[72] Benzon H.R.L., Lee S.C. (2016). Potential of wood vinegar in enhancing fruit yield and antioxidant capacity in tomato. Korean J. Plant Res..

[73] Fedeli R., Dichiara M., Carullo G., Tudino V., Gemma S., Butini S., Campiani G., Loppi S. (2024). Unlocking the potential of biostimulants in sustainable agriculture: Effect of wood distillate on the nutritional profiling of apples. Heliyon.

[74] Kårlund A., Salminen J.P., Koskinen P., Ahern J.R., Karonen M., Tiilikkala K., Karjalainen R.O. (2014). Polyphenols in strawberry (*Fragaria× ananassa*) leaves induced by plant activators. J. Agric. Food Chem..

[75] Petruccelli R., Bonetti A., Traversi M.L., Faraloni C., Valagussa M., Pozzi A. (2015). Influence of biochar application on nutritional quality of tomato (*Lycopersicon esculentum*). Crop Pasture Sci..

[76] Simiele M., Argentino O., Baronti S., Scippa G.S., Chiatante D., Terzaghi M., Montagnoli A. (2022). Biochar enhances plant growth, fruit yield, and antioxidant content of cherry tomato (*Solanum lycopersicum* L.) in a soilless substrate. Agriculture.

[77] Srivastava A., Akoh C.C., Yi W., Fischer J., Krewer G. (2007). Effect of storage conditions on the biological activity of phenolic compounds of blueberry extract packed in glass bottles. J. Agric. Food Chem..

[78] Zheng W., Wang S.Y. (2003). Oxygen radical absorbing capacity of phenolics in blueberries, cranberries, chokeberries, and lingonberries. J. Agric. Food Chem..

[79] Aleklett K., Rosa D., Pickles B.J., Hart M.M. (2022). Community assembly and stability in the root microbiota during early plant development. Front. Microbiol..

[80] Normander B.O., Prosser J.I. (2000). Bacterial origin and community composition in the barley phytosphere as a function of habitat and presowing conditions. Appl. Environ. Microbiol..

[81] Zhang R., Dai W., Yao Z., Zhao C., An X. (2014). Effects of wood vinegar on the soil microbial characteristics. J. Chem. Pharm. Res..

[82] Sivaram A.K., Panneerselvan L., Mukunthan K., Megharaj M. (2022). Effect of pyroligneous acid on the microbial community composition and plant growth-promoting bacteria (PGPB) in soils. Soil Syst..

[83] https://biodea.bio/shop-biodea/.

[84] Paltineanu C., Coman M., Nicolae S., Ancu I., Calinescu M., Sturzeanu M., Chitu E., Ciuciu M., Nicola C. (2018). Root system distribution of highbush blueberry crops of various ages in medium-textured soils. Erwerbs-Obstbau.

[85] Ali A., Santoro P., Mori J., Ferrante A., Cocetta G. (2024). Effect of UV-B elicitation on spearmint’s (*Mentha spicata* L.) morpho-physiological traits and secondary metabolites production. Plant Growth Regul..

[86] Khalid M.F., Jawaid M.Z., Nawaz M., Shakoor R.A., Ahmed T. (2024). Employing titanium dioxide nanoparticles as biostimulant against salinity: Improving antioxidative defense and reactive oxygen species balancing in eggplant seedlings. Antioxidants.

[87] Martelli F., Cirlini M., Lazzi C., Neviani E., Bernini V. (2020). Edible seaweeds and spirulina extracts for food applica-tion: In vitro and in situ evaluation of antimicrobial activity towards foodborne pathogenic bacteria. Foods.

[88] Chiancone B., Guarrasi V., Leto L., Del Vecchio L., Calani L., Ganino T., Galaverni M., Cirlini M. (2023). Vitro-derived hop (*Humulus lupulus* L.) leaves and roots as source of bioactive compounds: Antioxidant activity and polyphenolic profile. Plant Cell Tissue Organ Cult. (PCTOC).

[89] Schoebitz M., López M.D., Serri H., Aravena V., Zagal E., Roldán A. (2019). Characterization of bioactive compounds in blueberry and their impact on soil properties in response to plant biostimulants. Commun. Soil Sci. Plant Anal..

[90] Nazeer S., Agosti A., Del Vecchio L., Leto L., Di Fazio A., Hadj Saadoun J., Levante A., Lazzi C., Cirlini M., Chiancone B. (2024). Assessment of Fermented Kiwifruit on Morpho-Physiological and Productive Performances of *Fragaria* spp. Plants, Grown Under Hydroponic Conditions. J. Sustain. Agric. Environ..

