# Supplementary material for: Combined Effects of Biochar and Wood Distillate on Growth, Yield, and Fruit Quality of Soilless-Grown Highbush Blueberry Plants (Vaccinium corymbosum L.)
Source: Plants (Basel). 2025 Dec 11;14(24):3773. doi: 10.3390/plants14243773 (PMC12737149; doi:10.3390/plants14243773)

Supplementary Tables

Table S1: Effect of the "BC Treatment" and the "WD Treatment" on Fv/Fm, Flv and SPAD and Performance Index (PI) at harvest time .

Treatment	WD Presence	Flv	SPAD	Fv/Fm	PI
BC0		1.39±0.05	46±2	0.789±0.01	3.9±0.3
BC5	0WD	1.58±0.03	46±2	0.787±0.01	4.6±0.6
BC10		1.48±0.06	44±3	0.783±0.01	3.9±0.4
BC0		1.47±0.05	44±2	0.789±0.01	5.2±0.5
BC5	WD	1.46±0.03	45±2	0.780±0.01	5.3±0.7
BC10		1.46±0.05	44±2	0.797±0.01	5.9±0.5
Statistical analysis of the factors					
BC Treatment (BC)		0.179	0.974	0.886	0.797
WD Treatment (WD)		0.297	0.530	0.718	0.002
BC × WD		0.678	0.432	0.706	0.234

Statistical analysis was performed using two-way ANOVA at harvest time followed by Tukey's post-hoc test ($p \leq 0.05$). Results are expressed as mean ± standard error.
Abbreviations: BC0: 100%Coconut Fiber-CF; BC5: 5% of biochar and 95% of CF (v/v); BC10: 10% of biochar and 90% of CF (v/v); 0WD: without wood distillate, WD: treatment with wood distillate. PI: Performance Index, Fv/Fm: value to describe variations in the photonic efficiency of Photosystem II, Flv: Flavonols, SPAD: indirect measurement of chlorophyll content.

Supplementary Figures

Figure S1: Effect of "BC Treatment", "WD Treatment" and "Harvest date" on blueberry fruit weight. Three-way ANOVA, Tukey's test, p=0.002. Within each BC treatment and WD treatment, per harvest date, different letters indicate statistically different values.

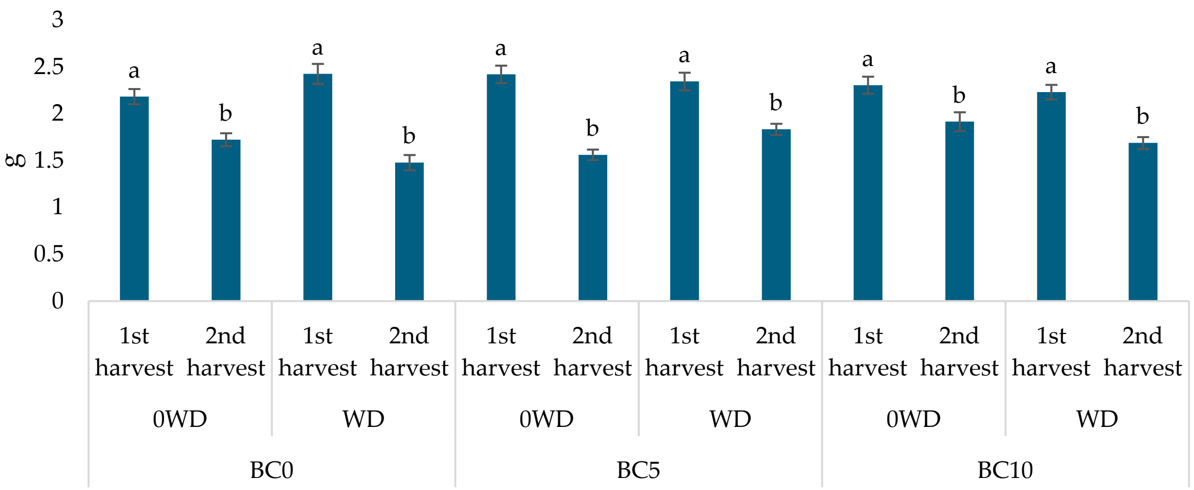

Figure S2: Effect of "BC Treatment", "WD Treatment" and "Harvest date" on blueberry fruit caliper. Three-way ANOVA, Tukey's test, p<0.001. Within each BC treatment and WD treatment, per harvest date, different letters indicate statistically different values.

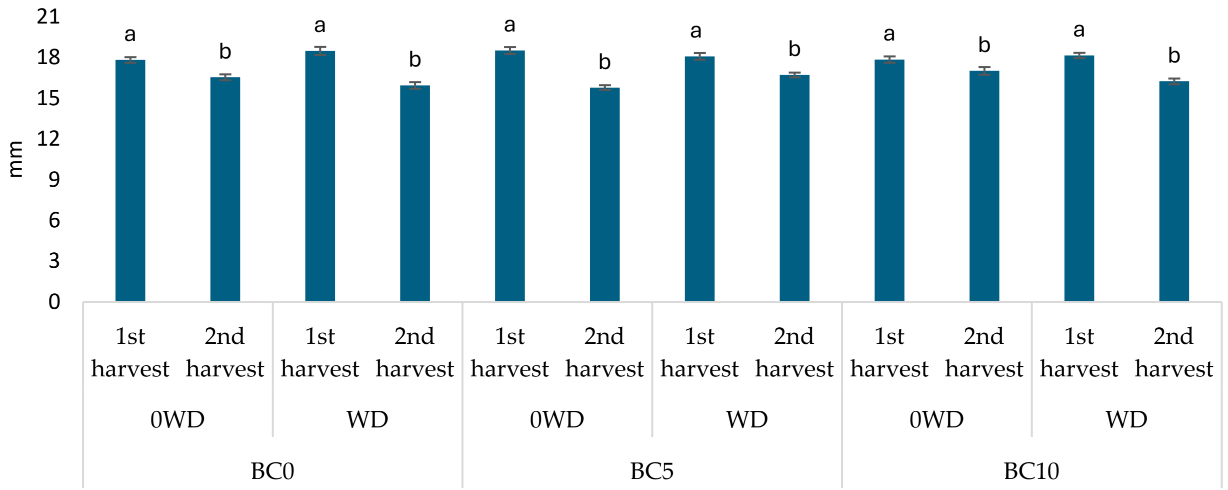

Figure S3: Effect of WD Presence and Harvest date on blueberry fruit Total Soluble Solids. Three-way ANOVA, Tukey’s test, p=0.024. Within each WD treatment, per harvest date, different letters indicate statistically different values.

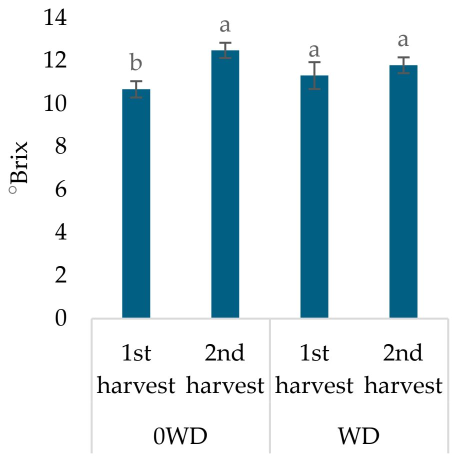

Figure S4: Effect of “BC Treatment”, “WD Treatment” and “Harvest date” on blueberry fruit acidity expressed as % citric acid. Three-way ANOVA, Tukey’s test, p=0.036. Within each BC treatment and WD treatment, per harvest date, different letters indicate statistically different values.

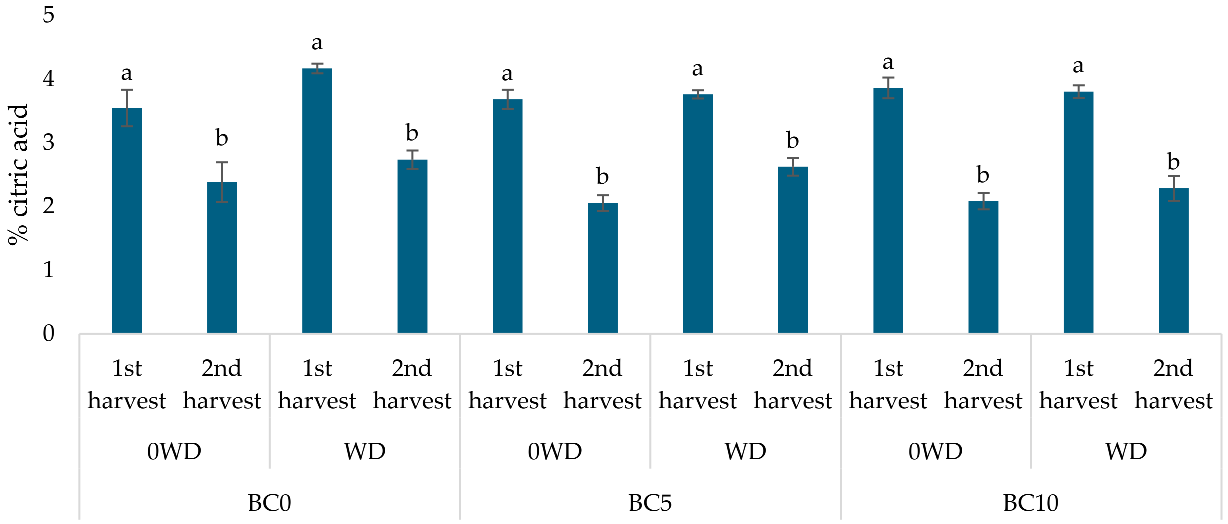

Figure S5: Effect of "BC Treatment" and "Harvest date" on blueberry fruit TSS/TA. Three-way ANOVA, Tukey's test, p<0.001. Within each BC treatment and WD treatment, per harvest date, different letters indicate statistically different values.

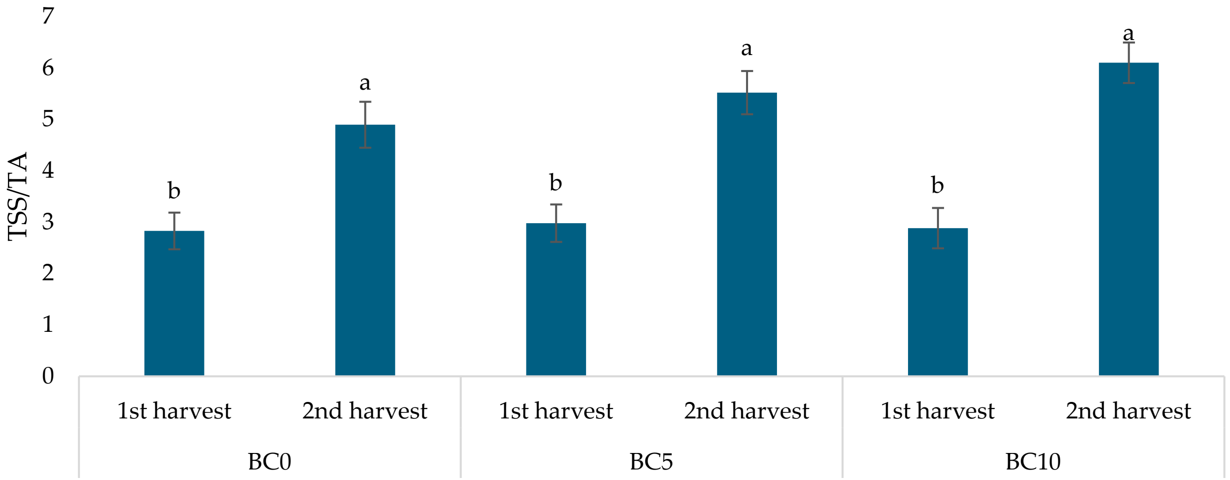

Figure S6: Effect of "BC Treatment", "WD Treatment" and "Harvest date" on blueberry fruit Total Phenolic Content . Three-way ANOVA, Tukey's test, p=0.012. Within each BC treatment and WD treatment, per each harvest date, different letters indicate statistically different values.

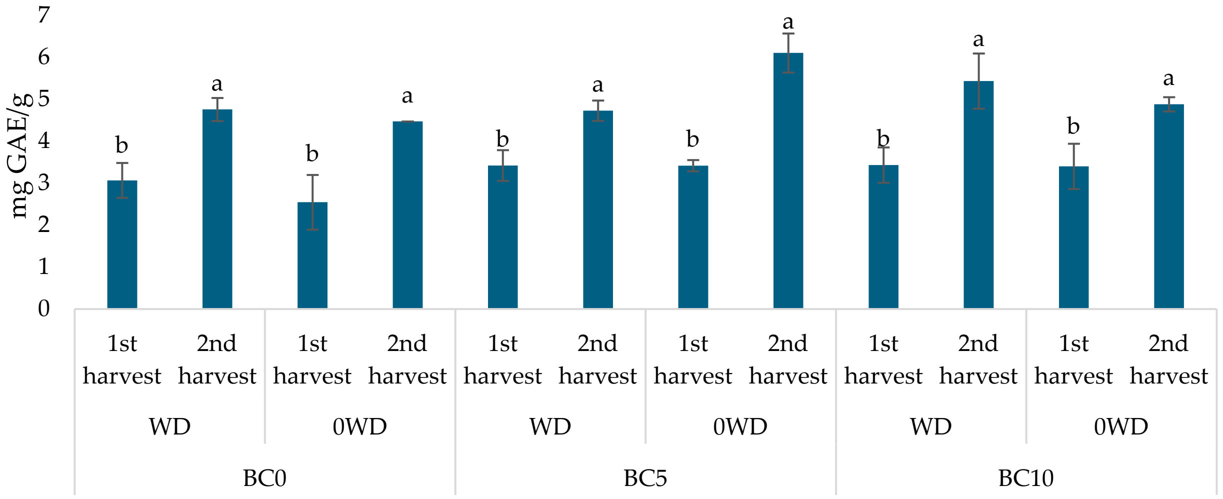

Figure S7: Effect of “BC Treatment”, “WD Treatment” and “Harvest date” on blueberry fruit Antioxidant Activity expressed as mM TEAC. Three-way ANOVA, Tukey’s test, p<0.001. Within each BC treatment and WD treatment, per harvest date, different letters indicate statistically different values.

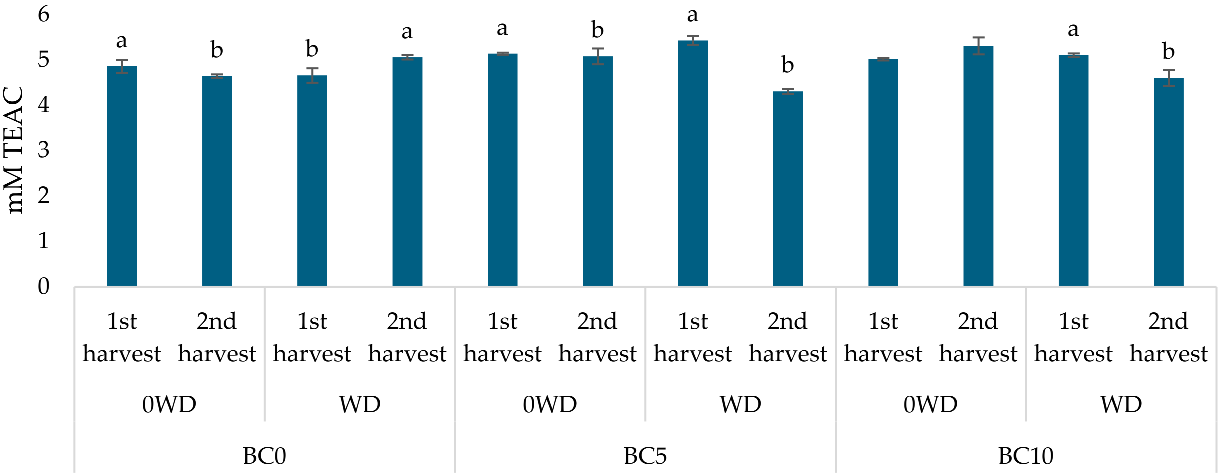

Figure S8: Effect of “BC Treatment”, “WD Treatment” and “Harvest date” on blueberry fruit Anthocyanins content expressed as mg/100g. Three-way ANOVA, Tukey’s test, p=0.005. Within each BC treatment and WD treatment, per harvest date, different letters indicate statistically different values.

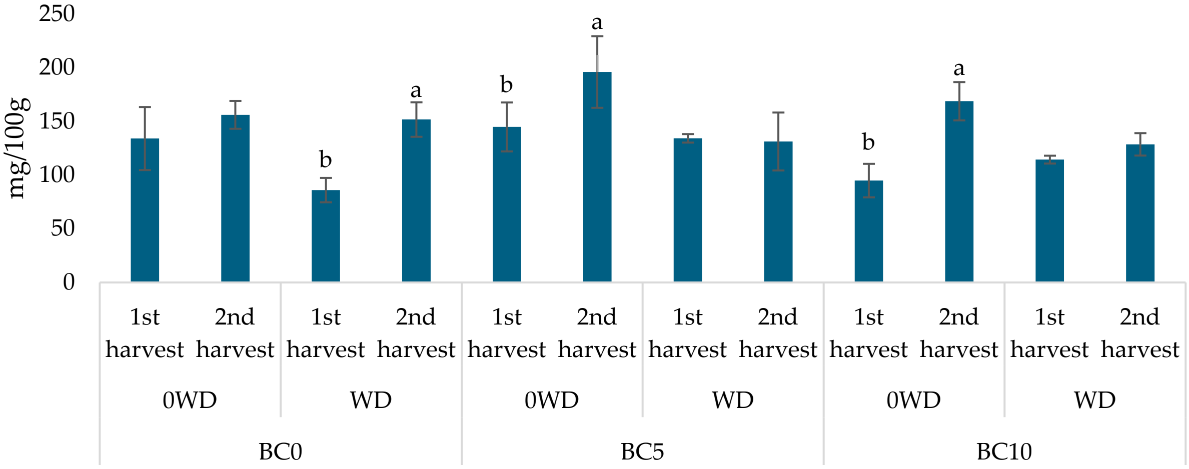

Figure S9: Experimental site at "I mirtilli di Zeno" farm (Farm SABIBI) in Ponzano Veneto, Treviso, Italy. Images of the plants (a) at the vegetative stage, when biochar was applied, and (b) at the flowering stage, when wood distillate application began.

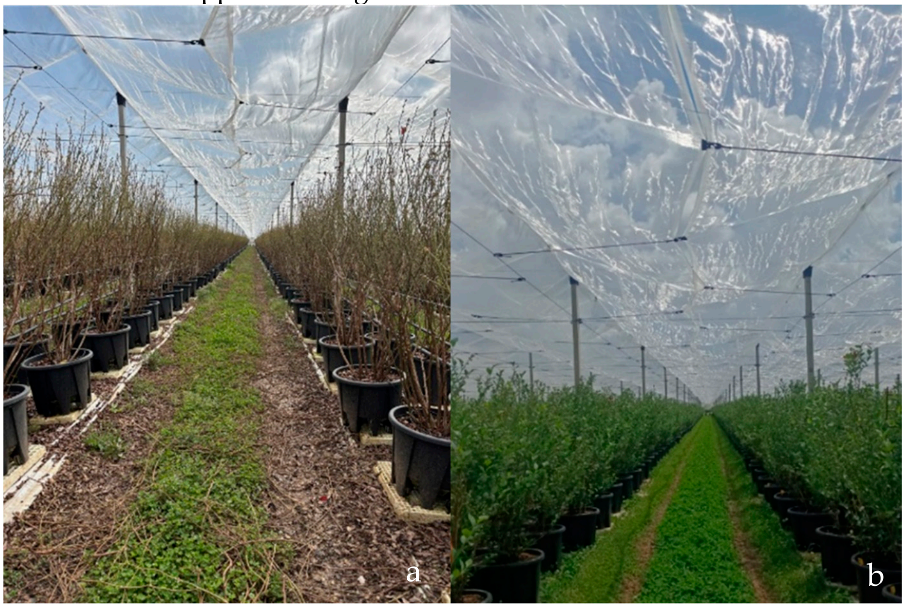

Figure S10: Spatial layout of the experimental area and randomized block design.The six treatments (BC0, BC0WD, BC5, BC5WD, BC10, BC10WD; 15 plants per treatment, 90 plants in total) were randomly assigned following a randomized block design. Colored circles indicate treatment-specific plants, whereas grey circles represent surrounding non-experimental plants.

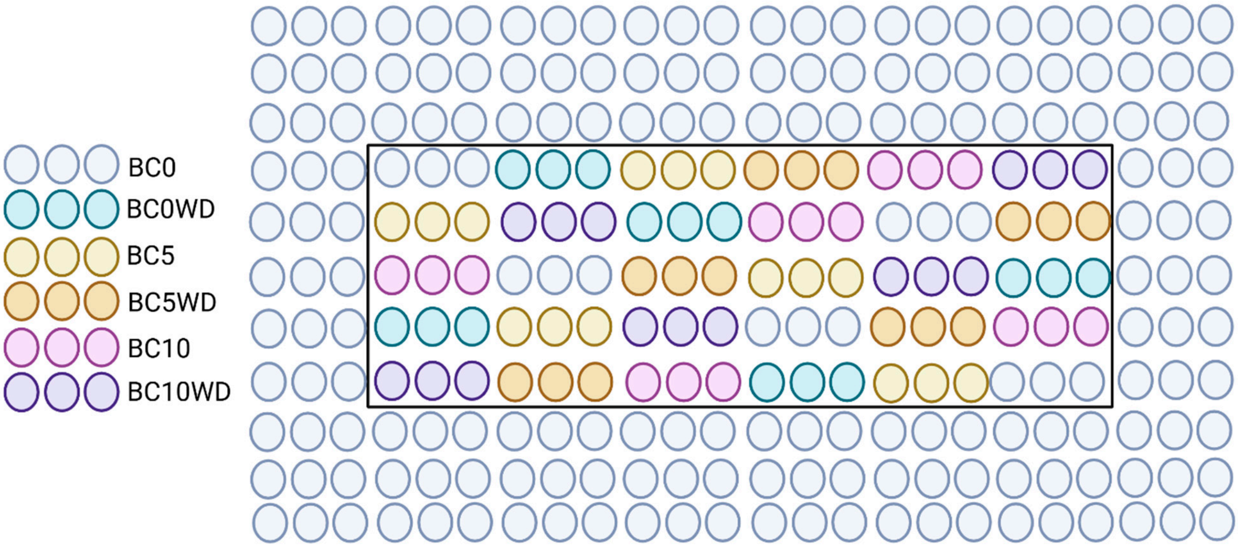

Figure S11: Triangular spacing scheme used in the experimental design for blueberry plants (Created in BioRender. Agosti, A. (2024) https://BioRender.com/k88m035).

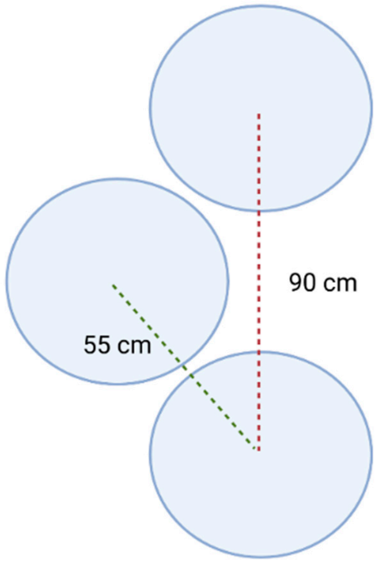

Supplement: Supplementary file 1 [file plants-14-03773-s001.zip › plants-3981111-supplementary.pdf]
